# Exploring a novel GH13_5 α-amylase from *Jeotgalibacillus malaysiensis* D5^T^ for raw starch hydrolysis

**DOI:** 10.1186/s13568-024-01722-3

**Published:** 2024-06-14

**Authors:** Nurfatini Radzlin, Mohd Shukuri Mohamad Ali, Kian Mau Goh, Amira Suriaty Yaakop, Iffah Izzati Zakaria, Ummirul Mukminin Kahar

**Affiliations:** 1 Malaysia Genome and Vaccine Institute, National Institutes of Biotechnology Malaysia, Jalan Bangi, 43000 Kajang, Selangor Malaysia; 2 Enzyme and Microbial Technology Research Centre, Faculty of Biotechnology and Biomolecular Sciences, Universiti Putra Malaysia, 43400 Serdang, Malaysia; 3 Department of Biochemistry, Faculty of Biotechnology and Biomolecular Sciences, Universiti Putra Malaysia, 43400 Serdang, Selangor Malaysia; 4 Enzyme Technology Laboratory, Institute Bioscience, Universiti Putra Malaysia, 43400 Serdang, Malaysia; 5 Department of Biosciences, Faculty of Science, Universiti Teknologi Malaysia, 81310 Skudai, Johor Malaysia; 6 School of Biological Sciences, Universiti Sains Malaysia, 11800 Minden, Pulau Pinang Malaysia

**Keywords:** α-Amylase, *Caryophanaceae*, Glycoside hydrolase family 13 subfamily 5, *Jeotgalibacillus*, Marine bacteria, Raw starch hydrolysis

## Abstract

α-Amylase plays a crucial role in the industrial degradation of starch. The genus *Jeotgalibacillus* of the underexplored marine bacteria family *Caryophanaceae* has not been investigated in terms of α-amylase production. Herein, we report the comprehensive analysis of an α-amylase (AmyJM) from *Jeotgalibacillus malaysiensis* D5^T^ (= DSM28777^T^ = KCTC33550^T^). Protein phylogenetic analysis indicated that AmyJM belongs to glycoside hydrolase family 13 subfamily 5 (GH13_5) and exhibits low sequence identity with known α-amylases, with its closest counterpart being the GH13_5 α-amylase from *Bacillus* sp. KSM-K38 (51.05% identity). Purified AmyJM (molecular mass of 70 kDa) is stable at a pH range of 5.5–9.0 and optimally active at pH 7.5. The optimum temperature for AmyJM is 40 °C, where the enzyme is reasonably stable at this temperature. Similar to other α-amylases, the presence of CaCl_2_ enhanced both the activity and stability of AmyJM. AmyJM exhibited activity toward raw and gelatinized forms of starches and related α-glucans, generating a mixture of reducing sugars, such as glucose, maltose, maltotriose, maltotetraose, and maltopentaose. In raw starch hydrolysis, AmyJM exhibited its highest efficiency (51.10% degradation) in hydrolyzing raw wheat starch after 3-h incubation at 40 °C. Under the same conditions, AmyJM also hydrolyzed tapioca, sago, potato, rice, and corn raw starches, yielding 16.01–30.05%. These findings highlight the potential of AmyJM as a biocatalyst for the saccharification of raw starches, particularly those derived from wheat.

**Supplementary Information:**

The online version contains supplementary material available at 10.1186/s13568-024-01722-3.

## Key points

- A novel GH13_5 α-amylase (AmyJM) from *Jeotgalibacillus malaysiensis* D5^T^ was successfully expressed, purified, and biochemically characterized.
- AmyJM hydrolyzed raw starches from wheat, tapioca, sago, potato, rice, and corn at 40 °C, forming mixtures of reducing sugars.
- The properties of AmyJM suggest that the enzyme is potentially applicable in direct raw starch degradation processes.

**Supplementary Information:**

The online version contains supplementary material available at 10.1186/s13568-024-01722-3.

## Introduction

α-Amylase (EC 3.2.1.1) is an amylolytic enzyme that randomly cleaves the α-1,4 glycosidic bonds in starch and related α-glucans, generating malto-oligosaccharides of varying lengths (Farias et al. 2021). Based on the Carbohydrate-Active enZYmes (CAZy) database classification, α-amylases are categorized within glycoside hydrolase (GH) families GH13, GH57, GH119, and GH126 (Drula et al. 2022). Notably, GH13 represents the largest family of amylolytic enzymes and is classified into 47 subfamilies (Janeček and Svensson 2022).

GH13 α-amylases share a standard structure consisting of three main domains (Miao et al. 2018). Domain A, located at the N-terminus, is the catalytic domain and adopts a TIM-barrel structure housing the catalytic triad composed of Asp, Glu, and Asp, along with conserved sequence regions (CSRs) I–VIII (Janeček and Svensson 2022). Domain B is an extended loop region that protrudes out of the catalytic domain, whereas domain C adopts a β-sandwich fold and is positioned at the C-terminus (Janeček et al. 2014; Zhang et al. 2017).

GH13 α-amylases commonly bind to polysaccharides (i.e., starch) through dedicated binding sites outside the enzyme’s active site region (Janeček and Svensson 2022). These additional binding sites can be found on carbohydrate-binding modules (CBMs), autonomous functional and folding domains, or surface binding sites (SBSs) positioned on the enzyme’s surface at either the catalytic domain or other domains (Cockburn et al. 2014; Janeček et al. 2019). Among the 101 established CBM families cataloged in the CAZy database (Drula et al. 2022), examples of CBM possessed by GH13 α-amylases include CBM20, CBM25, and CBM26 (Janeček et al. 2019). In contrast, certain GH13 α-amylases exhibit SBS, such as those found in subfamilies GH13_1, 2, 3, 4, 5, 6, 7, 8, 9, 10, 11, 14, 21, 24, and 31 (Cockburn et al. 2014).

Starch, the most abundant natural polysaccharide, consists of amylose and amylopectin (Mathobo et al. 2021). Amylose is a linear polymer formed by glucose units linked via α-1,4 glycosidic bonds, whereas amylopectin is a highly branched polymer consisting of glucose residues connected by α-1,4 and α-1,6 glycosidic bonds (Chakraborty et al. 2020). Conventional industrial starch processing involves two main stages: liquefaction and saccharification. Starch granules are gelatinized at a high temperature in the liquefaction process, typically around 100 °C (Li et al. 2023). In the subsequent saccharification process, a combination of several GH13 enzymes (i.e., α-amylase, type I pullulanase, and glucoamylase) is used to further degrade the starch slurry to produce sugar syrups (e.g., glucose and maltose) (Li et al. 2023), which serve as essential raw materials for various applications, including food products (e.g., beverages and baking) and non-food industries (e.g., biofuels) (Farooq et al. 2021). 

However, the energy-intensive nature of the starch gelatinization process significantly inflates the production cost of starch-based products (Li et al. 2023). Enzymatic degradation of raw starch granules below the gelatinization temperature can simplify the entire starch conversion process (Božić et al. 2017). Certain amylolytic enzymes, such as raw starch-degrading α-amylases, can achieve this. Using raw starch-degrading α-amylases could reduce energy consumption by approximately 15% compared with traditional physical or chemical processes (Sun et al. 2010). Therefore, raw starch-degrading α-amylases have potential industrial application as a non-thermal processing strategy to the traditional heating process in starch liquefaction (Božić et al. 2020; Fang et al. 2019; Slavić et al. 2023). 

Marine bacteria can be used to identify raw starch-degrading α-amylases as alternatives to the current industrial starch processing methods (Goh et al. 2019; Zhang et al. 2017). *Caryophanaceae* is a bacterial family in marine ecosystems (Gupta and Patel 2020) that comprises 20 genera archived in the List of Prokaryotic Names with Standing in Nomenclature (LPSN) database (Parte et al. 2020). *Jeotgalibacillus* is one of the less explored genera within *Caryophanaceae.* This genus has only one report on an industrially relevant enzyme, GH1 β-glucosidase (Liew et al. 2018). *Jeotgalibacillus malaysiensis* D5^T^ (= DSM28777^T^ = KCTC33550^T^) was isolated from a beach in Johor, Malaysia (Yaakop et al. 2015). Genome analyses of this bacterium revealed the presence of at least six putative genes encoding industrially important amylolytic enzymes from the GH13 family (Goh et al. 2015; Yaakop et al. 2015). In the present study, we describe the results of bioinformatics analysis, purification, and biochemical characterization of a GH13_5 α-amylase (denoted as AmyJM) originating from *J. malaysiensis* D5^T^. Our findings indicate that AmyJM can hydrolyze raw starches, suggesting its potential as a candidate for application in the direct bioconversion of raw starches to sugar syrups. To the best of our knowledge, this is the first report on an α-amylase from *Jeotgalibacillus* spp.

## Materials and methods

### Reagents and chemicals

Unless otherwise stated, the chemicals were of analytical and molecular grade and purchased from Merck KGaA (Darmstadt, Germany). Kanamycin sulfate was obtained from Calbiochem (San Diego, CA, USA). Soluble starch from potatoes was purchased from Kanto Chemical Co., Inc. (Tokyo, Japan). Tapioca and sago starches were of food grade and procured from THC Sdn. Bhd. (Penang, Malaysia). Amylose from potato and β-limit dextrin from maize were purchased from Megazyme (County Wicklow, Ireland, UK). High-grade (≥99% purity) glucose (G1), maltose (G2), maltotriose (G3), maltotetraose (G4), maltopentaose (G5), maltohexaose (G6), and maltoheptaose (G7) were obtained from Elicityl (Crolles, France).

### Bioinformatics analysis of AmyJM

A putative α-amylase amino acid sequence was derived from the annotated complete genome of *J. malaysiensis* D5^T^. The α-amylase is designated as AmyJM, with accession number A0A0B5ARF3 in the UniProtKB database (Consortium 2023). Based on the dbCAN3 CAZy meta server (Zheng et al. 2023) family classification, AmyJM was classified in the subfamily GH13_5 (accessed on February 1, 2024). Sequences homologous to AmyJM were extracted from the CAZy database (Drula et al. 2022), focusing on biochemically characterized and crystallized α-amylases of subfamily GH13_5 (available as of February 1, 2024). Additional α-amylase sequences were obtained by NCBI BLASTp searches against the “non-redundant protein sequences (nr)” database. Multiple protein sequence alignments were performed using the Clustal Omega web server (Madeira et al. 2022). Phylogenetic trees were generated by the neighbor-joining method using Molecular Evolutionary Genetic Analysis (MEGA v.11.0.13) with a bootstrap value of 1,000 replicates (Tamura et al. 2021). Sequence logos of eight conserved sequence regions (CSRs) were created using the WebLogo3 online server (Crooks et al. 2004). Putative protein domains were predicted using the InterProScan v.5.56-89.0 online server (Paysan-Lafosse et al. 2023). The 3D homology model of AmyJM was retrieved from the AlphaFoldDB protein structure database (model number: AF-A0A0B5ARF3-F1) (Varadi et al. 2023); the quality of the AmyJM model was verified using the Structural Analysis and Verification Server (SAVES v.6.0). The AmyJM homology model was viewed and analyzed using PyMol v11 (Schrödinger, New York, USA). Default parameters were used for all software tools unless otherwise specified.

### Expression and purification of recombinant AmyJM

The *amyJM* gene was synthesized by the GenScript Corporation (Piscataway, NJ, USA). The synthetic gene was cloned into pET-28a(+) (Novagen/Merck KGaA) using the *Bam*HI and *Xho*I restriction sites. The pET-28a(+) construct was transformed into *Escherichia coli* BL21(DE3) (New England BioLabs, Ipswich, MA, USA). To express AmyJM, recombinant *E. coli* BL21(DE3) was grown on Luria–Bertani (LB) agar (pH 7.0) supplemented with 50 µg/mL kanamycin sulfate (kan) at 37 °C for 24 h. A single colony of recombinant *E. coli* BL21(DE3) was inoculated into 50 mL of LB/kan medium (pH 7.5) in a 250 mL flask and cultured under shaking at 200 rpm at 37 °C for 24 h. A 2 mL inoculum (equivalent to 1% v/v) was transferred into 200 mL of fresh LB/kan medium (pH 7.5) in a 1 L flask and incubated at 37 °C, 200 rpm. At periodic intervals, culture medium absorbance at 600 nm (*A*_600_) was recorded using an Ultrospec 2100 *pro* UV/Visible Spectrophotometer (Cytiva, Marlborough, MA, USA). When the *A*_600_ reached 0.5, enzyme expression was induced by adding a final concentration of 0.4 mM isopropyl-β-D-thiogalactopyranoside and further incubation at 37 °C, 200 rpm for 4 h. Then, the culture was centrifuged at 5,000 × *g*, 4 °C for 10 min, and the cell pellet was collected. To obtain crude AmyJM, the pellet was lysed using a B-PER™ Bacterial Protein Extraction Reagent Kit (Thermo Fisher Scientific, Rockford, IL, USA), according to the manufacturer’s instructions. The cell-free lysate was dialyzed against 100 mM sodium phosphate buffer (pH 7.5) at 4 °C for 18 h using SnakeSkin dialysis tubing with a 10-kDa molecular weight cut-off, MWCO (Thermo Fisher Scientific).

The crude AmyJM was purified using a pre-packed 1 mL HisPur^™^ nickel-nitrilotriacetic (Ni-NTA) chromatography cartridge (Thermo Fisher Scientific). The cartridge was equilibrated with 20 mM sodium phosphate buffer, 300 mM NaCl, 55 mM imidazole, pH 7.4. The bound enzyme was eluted with a linear gradient of 55–300 mM imidazole. The active fractions were pooled and dialyzed against 100 mM sodium phosphate buffer (pH 7.5) at 4 °C for 18 h using SnakeSkin dialysis tubing, 10-kDa MWCO (Thermo Fisher Scientific). The purified AmyJM was used for subsequent analyses.

### Enzyme and protein assays

α-Amylase activity was determined using the 3,5-dinitrosalicylic acid (DNS) method (Miller 1959). A reaction mixture consisting of 0.1 mL of enzyme (5.0 U/mg; 1.0 mg/mL) and 0.9 mL of 1% (w/v) soluble starch in 100 mM sodium phosphate buffer (pH 7.5) was incubated at 40 °C for 15 min. DNS reagent (1 mL) was then added to the mixture, followed by boiling (100 °C) for 5 min. Subsequently, *A*_540_ was measured using the Ultrospec 2100 *pro* UV/Visible Spectrophotometer. As a control, an unreacted mixture was incubated and analyzed under the same conditions. Maltose was used as the assay standard. One unit (U) of α-amylase activity was defined as the amount of enzyme that generated 1 µmol of reducing sugar per minute per milliliter at 40 °C. The protein concentration was quantified using a PIERCE^™^ bicinchoninic acid (BCA) Protein Assay Kit (Thermo Fisher Scientific) with bovine serum albumin as the standard. The assays were performed at least in triplicate unless otherwise specified.

### Characterization of AmyJM

#### Gel electrophoresis and zymography

The molecular mass and purity of AmyJM were determined using 12% (v/v) sodium dodecyl sulfate-polyacrylamide gel electrophoresis (SDS-PAGE) analysis. Imperial^™^ Protein Stain (Thermo Fisher Scientific) was used to stain the protein bands, which were compared with Benchmark^™^ Protein Ladder (Life Technologies, Carlsbad, CA, USA) to estimate the molecular mass. Zymogram staining for the detection of AmyJM starch-degrading activity was performed as previously described (Yang et al. 2004), except that 1% (w/v) soluble starch was dissolved in 100 mM sodium phosphate buffer (pH 7.5) and incubated at 40 °C for 15 min.

#### Effects of pH, buffer, and temperature

The optimum pH for AmyJM was determined at 40 °C using the following buffers (100 mM each): glycine-HCl (pH 2.0–3.0), sodium acetate (pH 4.0–5.5), sodium phosphate (pH 6.0–7.5), Tris-HCl (pH 8.0–9.0), and glycine-NaOH (pH 10.0–11.0). To measure the pH stability of AmyJM, the enzyme was incubated in each buffer without substrate at 25 °C for 20 min, and residual activity was measured under standard assay conditions.

The effects of different buffers on AmyJM activity were determined by reacting the enzyme with soluble starch dissolved in five different buffers (100 mM each, pH 7.5): sodium phosphate, potassium phosphate, Tris-HCl, HEPES-NaOH, MOPS, at 40 °C, and measuring the residual activity.

The optimum temperature for AmyJM was evaluated at 10–90 °C in the optimum enzyme buffer (100 mM sodium phosphate buffer, pH 7.5). To investigate its thermostability, the enzyme was pre-incubated at different temperatures for 20 min without substrate, and residual activity was measured. The thermostability of AmyJM was further evaluated by pre-incubating the enzyme with or without 5 mM CaCl_2_ at 40–50 °C for 150 min, taking samples at periodic intervals, and measuring residual activity under standard assay conditions.

#### Kinetic parameters

The kinetic parameters were assessed by measuring maltose formation by AmyJM for different concentrations of soluble starch (2–40 mg/mL) in 100 mM sodium phosphate buffer (pH 7.5) at 40 °C. The values of the Michaelis constant (*K*_m_), maximum velocity (*V*_max_), and turnover number (*k*_cat_) of AmyJM were determined using the GraphPad Prism v.9.0.0 software (GraphPad Software Inc., La Jolla, CA, USA).

#### Effects of metal ions and chemical reagents

The influence of various additives on AmyJM activity was investigated using varying concentrations of chloride salts (5 and 10 mM each): calcium chloride, magnesium chloride, sodium chloride, potassium chloride, ammonium chloride, zinc chloride, copper (II) chloride, nickel (II) chloride, cobalt (II) chloride, manganese (II) chloride, and iron (III) chloride. Besides, effect of various chemical reagents on AmyJM activity was evaluated using (5 and 10 mM each): ethylenediaminetetraacetic acid (EDTA), urea, and β-mercaptoethanol, and (5% v/v and 10% v/v each): Triton X-100, Tween-20, Tween-80, dimethyl sulfoxide (DMSO), and sodium dodecyl sulfate (SDS). All the additives were added to the standard enzymatic assay and incubated at 40 °C in 100 mM sodium phosphate buffer (pH 7.5). Residual activity was measured, and enzyme activity without the additives was used as a reference (100%).

#### Analysis of reaction products from gelatinized substrate

Purified AmyJM was concentrated using an Amicon^®^ Ultra-15 (10-kDa MWCO) Centrifugal Filter Unit (Merck KGaA). The concentrated AmyJM was used in subsequent reaction product analysis. All substrates used in the analysis were gelatinized by boiling (100 °C) in 100 mM sodium phosphate buffer (pH 7.5) with continuous stirring for 10 min, followed by cooling in a water bath at 40 °C for 10 min. The hydrolytic ability of AmyJM was determined by separately incubating the concentrated AmyJM (40 U) with various gelatinized substrates (1% w/v each), including soluble starch, wheat starch, tapioca starch, sago starch, potato starch, rice starch, corn starch, pullulan, amylose, amylopectin, β-limit dextrin, glycogen, and β-cyclodextrin (β-CD). All reactions were conducted for 24 h in a water bath at 40 °C, shaking at 100 strokes per min. The reactions were stopped by boiling (100 °C) for 10 min. Insoluble particles were filtered through a 0.45-µm nylon membrane syringe filter (Millex-GN/Merck KGaA) and subjected to high-performance liquid chromatography with evaporative light-scattering detection (HPLC-ELSD) analysis.

The reaction products were analyzed using an Agilent 1260 Infinity HPLC system with an Agilent 1260 Infinity ELSD (Agilent Technologies, Santa Clara, CA, USA). The column employed was a 0.5-µm Zorbax carbohydrate analysis (NH_2_) column, 4.6 × 150 mm (Agilent Technologies). The column temperature was maintained at 30 °C. The ELSD nebulizer and evaporator temperatures were maintained at 30 °C, and the nitrogen gas flow was maintained at 1.6 L/min. Acetonitrile-water (75:25, v/v) was used as the mobile phase at a 1 mL/min flow rate. High-grade (≥ 99% purity) glucose (G1), maltose (G2), maltotriose (G3), maltotetraose (G4), maltopentaose (G5), maltohexaose (G6), and maltoheptaose (G7) were used as standards. Unreacted substrates were also injected under the same chromatographic conditions as controls.

### Raw starch degradation by AmyJM

#### Determination of starch amylose/amylopectin composition

The amylose/amylopectin ratios in wheat, tapioca, sago, potato, rice, and corn starches were determined using an Amylose/Amylopectin Assay Kit (Megazyme), according to the manufacturers’ instructions.

#### Adsorption, hydrolysis, and morphology of raw starch

The adsorption and hydrolytic abilities of AmyJM toward raw starches were assessed by incubating AmyJM (40 U) with 1% (w/v) of various raw starch granules (wheat, tapioca, sago, potato, rice, and corn) in 100 mM sodium phosphate buffer (pH 7.5) in a final volume of 5 mL. All reactions were conducted in a shaking water bath (40 °C, 100 strokes per min) for 3 h. After centrifugation at 12,000×g, 4 °C for 3 min, residual activities in the supernatants were measured under the standard assay conditions. As a control, an unreacted mixture was incubated and analyzed under the same conditions. The percentage adsorption was calculated using the following formula (Nisha and Satyanarayana 2015):

$$Percentage\, absorption\, (\%) = 100-[(C/C_{0})]\times100]$$

where *C* is the enzyme activity in the supernatant after binding, and *C*_0_ is the initial enzyme activity.

The degree of raw starch hydrolysis (*R*_h_) was defined using the following formula, (Shofiyah et al. 2020):

$$R_{h}\,(\%)=(A_{1}/A_{0})\times100.$$

where *A*_1_ is the total amount of sugars in the supernatant after the reaction, and *A*_0_ is the amount of raw starch before the reaction.

Field emission scanning electron microscopy (FESEM) was used to observe the raw starch granules before and after hydrolysis by AmyJM. The pellets recovered from the aforementioned reactions were treated as previously described (Fang et al. 2019). The samples were then mounted on a specimen holder using a silver plate, sputtered with gold, and viewed under a high-resolution FEI Quanta 650 FEG field emission scanning electron microscope (Thermo Fisher Scientific, Hillsboro, OR, USA) operated at 10 kV.

#### Analysis of the hydrolysis products

To analyze the ability of AmyJM to hydrolyze 13 different raw substrates, AmyJM (40 U) was mixed with 1% (w/v each) of soluble starch, wheat starch, tapioca starch, sago starch, potato starch, rice starch, corn starch, pullulan, amylose, amylopectin, β-limit dextrin, glycogen, or β-CD in 100 mM sodium phosphate buffer (pH 7.5). Subsequently, the reaction mixtures were incubated in a shaking water bath (40 °C, 100 strokes per min) for 24 h. The enzymatic reactions were stopped by boiling (100 °C) for 10 min, filtered through a 0.45-µm nylon-membrane syringe filter, and subjected to HPLC-ELSD under the aforementioned conditions. Non-reacted substrates served as controls.

### Statistical analysis

The enzymatic assays and HPLC-ELSD analyses were analyzed using SYSTAT v.12.02.00 software (Systat Software, San Jose, CA, USA). Student’s *t*-test yielded a probability value (*p* value) of < 0.05, confirming that the data were adequate to test all hypotheses.

## Results

### Bioinformatics analysis of AmyJM

**Fig. 1:**
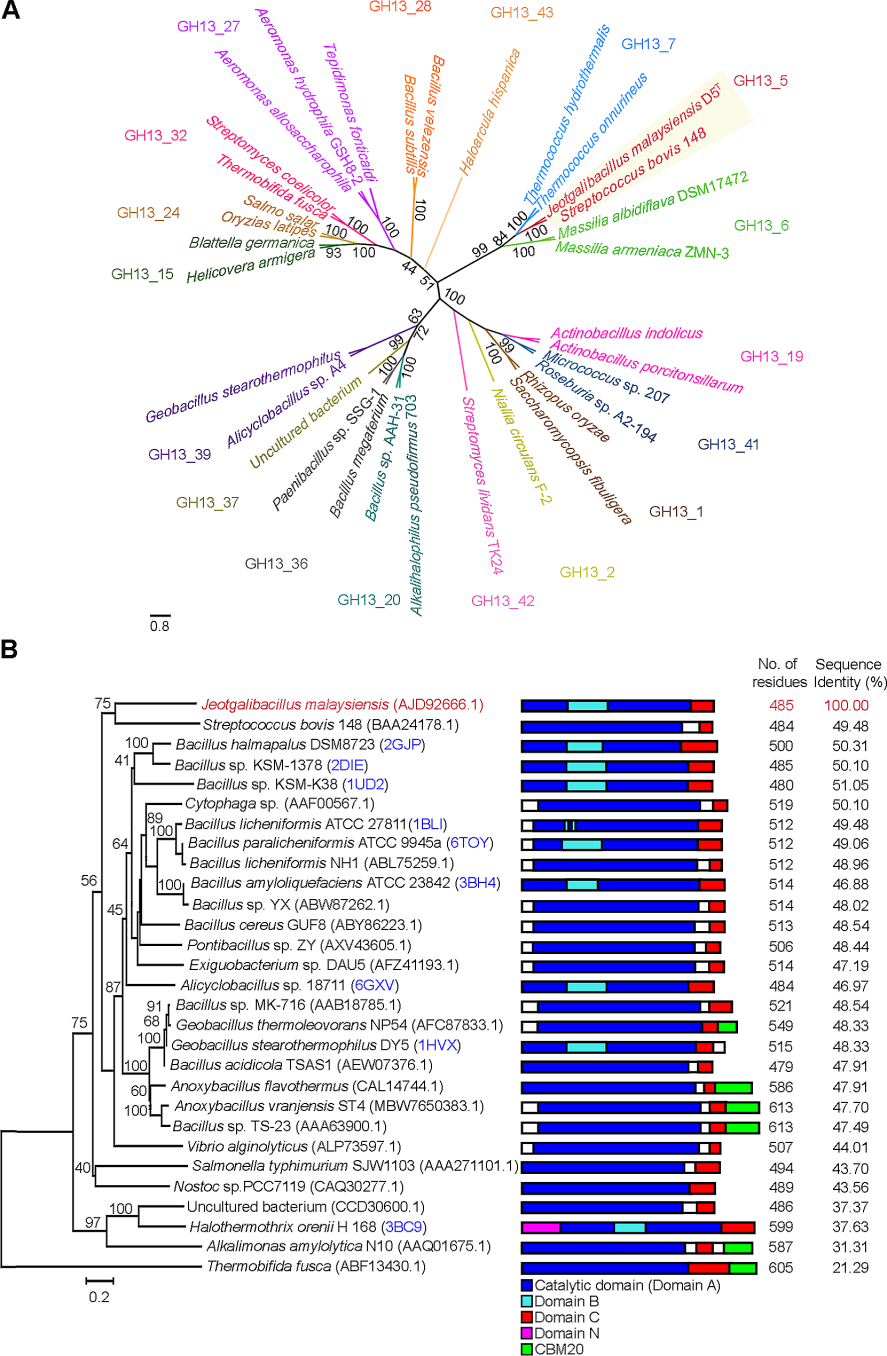
**A** Evolutionary tree of GH13 family bacterial α-amylases. The α-amylase from *Jeotgalibacillus malaysiensis* D5^T^ (AmyJM) was clustered in subfamily GH13_5. The α-amylase sequences were aligned using the Clustal Omega web server (Madeira et al. 2022). The tree was constructed using the neighbor-joining method in the Molecular Evolutionary Genetic Analysis (MEGA v.11.0.13) software (Tamura et al. 2021), with 1000 bootstrap replicates. The scale bar represents 0.8 nucleotide substitution per site. ^T^Type strain. **B** Protein relationship tree of AmyJM and all 27 well-characterized bacterial α-amylases from subfamily GH13_5. Schematic representations of domain arrangements for the α-amylases are shown in the box. The NCBI accession numbers or PDB IDs (labeled in blue) are indicated in parentheses. The α-amylase from *Thermobifida fusca* belonging to subfamily GH13_32 was used as an out-group. Sequence identity (%) refers to amino acid sequence identity (%) of AmyJM with other GH13_5 bacterial α-amylases. The scale bar represents 0.2 amino acid substitution per site. ^T^Type strain

AmyJM is the sole GH13_5 α-amylase sequence curated in the CAZy database from the genus *Jeotgalibacillus.* The mature sequence of the *amyJM* gene (1,455 bp) encoding an α-amylase (485 amino acids) was retrieved from the complete genome of *J. malaysiensis* D5^T^. Figure 1A shows an evolutionary tree of bacterial α-amylases constructed using the protein sequences of AmyJM and representative members from each of the α-amylase GH13 subfamilies. The tree demonstrates that AmyJM is clustered with members from GH13 subfamily 5 (GH13_5). A separate tree was constructed to show the relationships between AmyJM and all 27 well-characterized bacterial α-amylases from subfamily GH13_5 (Fig. 1B). AmyJM demonstrated low protein sequence identity (31.31–51.05%) with its homologs from other genera (Fig. 1B), underscoring its novelty. The closest relative to AmyJM is the α-amylase from *Bacillus* sp. KSM-K38 (CAC39917.1), sharing only 51.05% sequence identity. AmyJM is notably distinct from other GH13_5 bacterial α-amylases, such as those from *Nostoc* sp. PCC7119 (CAQ30277.1; 43.56%), *Halothermothrix orenii* H 168 (ACL70573.1; 37.63%), and *Alkalimonas amylolytica* N10 (AAQ01675.1; 31.31%) (Reyes-Sosa et al. 2010; Tan et al. 2008; Wang et al. 2006) (Fig. 1B).

Based on a multiple sequence alignment of AmyJM and all 27 well-characterized GH13_5 bacterial α-amylases (Additional file 1: Figs. S1, S2, and S3), AmyJM consists of eight CSRs (I–VIII) present in all GH13_5 bacterial α-amylases. The putative catalytic machineries of AmyJM were identified as D233, E263, and D330, identical to the active-site residues of GH13_5 α-amylases. These residues were located in CSR II, III, and IV, respectively (Additional file 1: Fig. S1). All CSRs and putative catalytic residues of AmyJM were found in domain A (the catalytic domain).

**Fig. 2:**
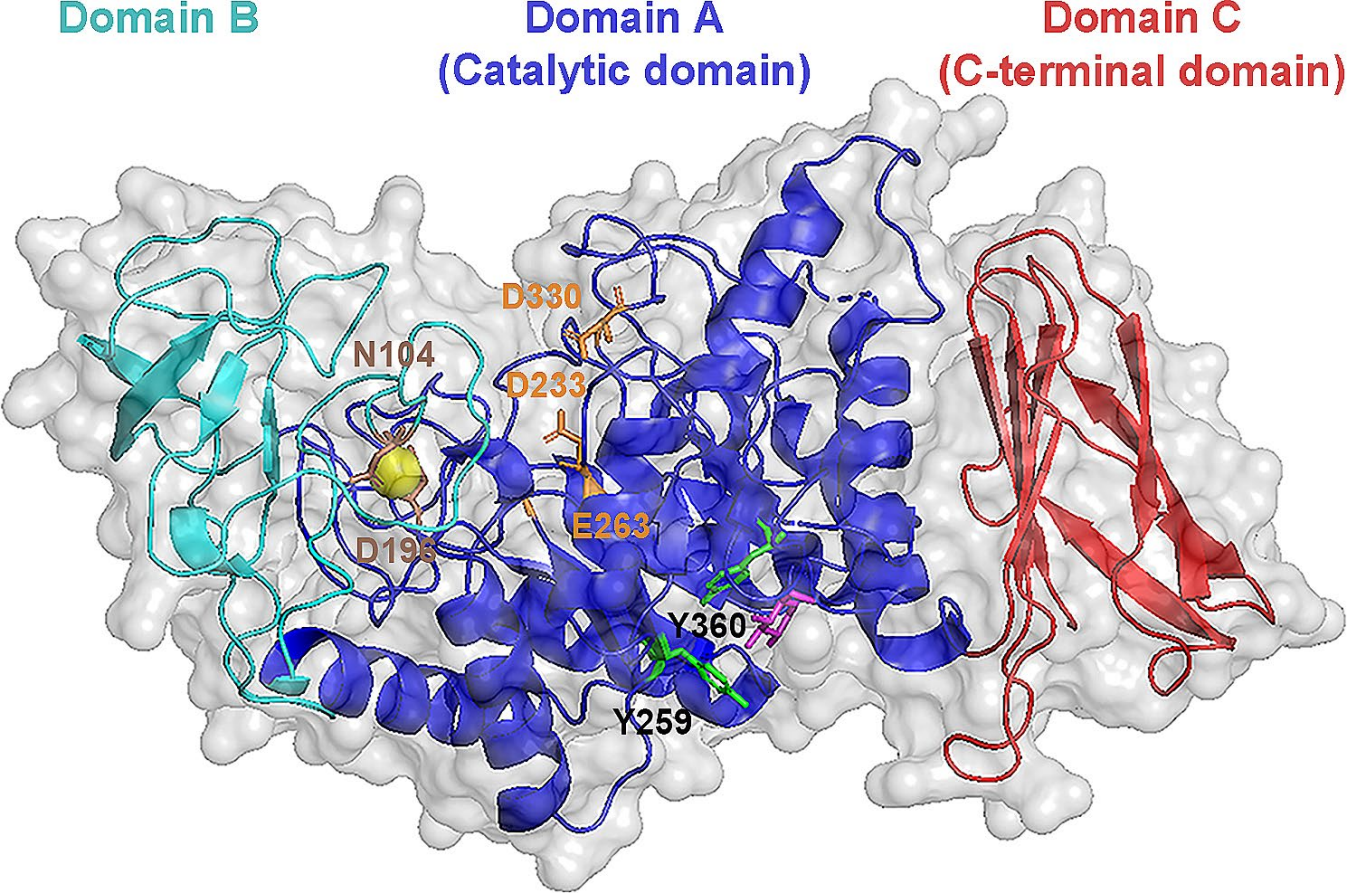
3D homology model of AmyJM. Putative catalytic sites (D233, E263, and D330) of AmyJM are shown in orange. The calcium ion is shown as a sphere. Putative calcium-interacting residues (N104 and D196) are shown in brown. The putative surface-binding site (residue pair Y259 and Y360) of AmyJM is shown in green. The glucose (G1) molecule in AmyJM is shown in pink

The high-quality model of AmyJM generated by the AlphaFoldDB protein structure database revealed that 99.7% of its total residues resided within favored or allowed regions of the Ramachandran plot. The N-terminus of AmyJM comprises domain A (residues M1–G108; E209–Y396) (Fig. 2 and Additional file 1: Fig. S1), which folds into a catalytic TIM-barrel structure featuring eight alternating β-strands and α-helices. AmyJM also possesses domain B (residues A109–P208), which extends outward from the TIM-barrel complex. This domain consists of a long loop connected to a β-strand and an α-helix from domain A. The C-terminus of AmyJM incorporates domain C (residues G397–E485), forming a β-sandwich structure. Notably, a calcium ion is positioned between domains A and B (Fig. 2). Residue N104 in domain A and D196 in domain B are identified as putative residues that interact with the calcium ion (Additional file 1: Fig. S2). Both residues are identical to their counterparts in the BHA X-ray structure (N106 and D199) (Lyhne-Iversen et al. 2006).

Within subfamily GH13_5 bacterial α-amylases, SBSs have been identified in the crystal structures of BHA (PDB ID: 2GJP) and those from *Bacillus paralicheniformis* ATCC 9945a (*Bli*Amy; 6TOY) and *Halothermothrix orenii* H 168 (AmyB; 3BC9), which these SBSs reportedly interact with sugar molecules (e.g., glucose and maltose) (Božić et al. 2020; Lyhne-Iversen et al. 2006; Tan et al. 2008). Sequence alignment and structure superimposition of AmyJM with BHA, *Bli*Amy, and AmyB suggested that AmyJM has one potential SBS (Fig. 2 and Additional file 1: Fig. S4). The putative SBS of AmyJM is formed by residues Y259 and Y360, which correspond to the *Bli*Amy SBS (residues F257 and Y358) (Božić et al. 2020). The putative SBS may interact with a glucose molecule positioned at the bottom of the AmyJM 3D model (Fig. 2).

### Characterization of AmyJM

**Fig. 3:**
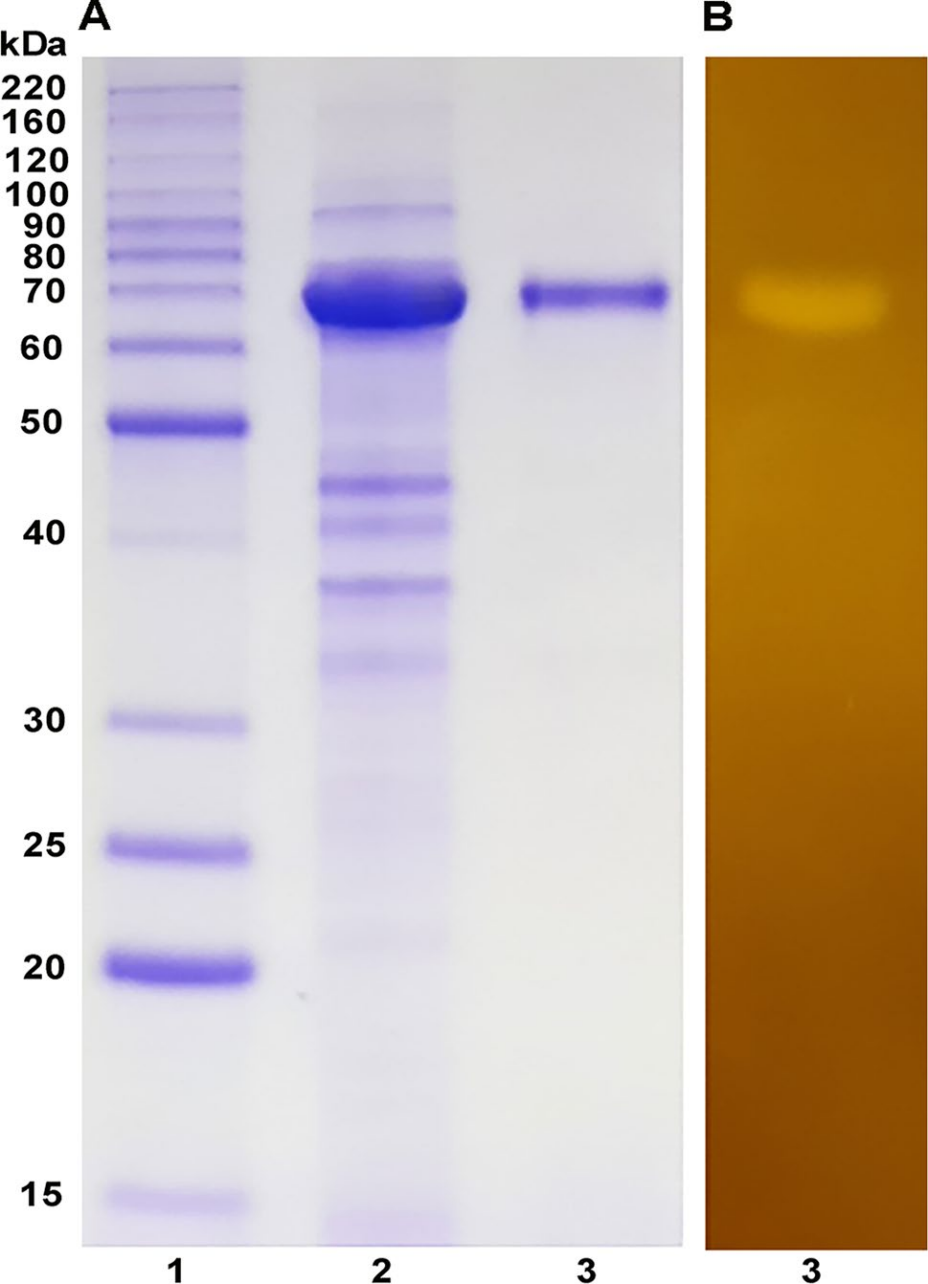
**A** SDS-PAGE (12% v/v) of AmyJM. **B** Zymogram of the amylolytic activity of AmyJM. Lane 1: molecular mass protein marker (BenchMark™ Protein Ladder), lane 2: crude enzyme, and lane 3: purified AmyJM

Table 1 presents a comparative summary of the characteristics of AmyJM and all 27 well-studied GH13_5 bacterial α-amylases. In the present study, recombinant AmyJM was purified to homogeneity by a single-step purification using a Ni-NTA affinity chromatography column. As shown in Fig. 3A, SDS-PAGE analysis indicated the high purity of AmyJM, with a molecular mass of approximately 70 kDa. The purified sample produced a clear band in zymogram analysis, demonstrating the starch-degrading capability of AmyJM (Fig. 3B).

**Table 1:** Purified GH13_5 bacterial α-amylases

Source	MM (kDa)	T_opt_ (°C)	pH_opt_	^a^Reaction product													References
				SS	WS	TS	SA	PS	RS	CS	P	AM	AP	D	G	β-CD	
				RSS	RWS	RTS	RSA	RPS	RRS	RCS	RP	RAM	RAP	RD	RG	Rβ-CD	
Degrade raw and gelatinized starches																	
*Jeotgalibacillus malaysiensis* D5^T^	70	40	7.5	G2, G6	G1–G5	G3, G4, G6	G2, G3 G4, G6	G2, G3, G4, G6	G1	G2, G6	NR	G1, G2, G5	G1–G5	G1–G4	G1–G3	–	This study
				G2, G6	G2, G4, G5	G3, G6	G1, G2, G6	G1, G6	G1	G2, G6	NR	G1, G2, G5	G1, G3	G1–G4	G5	–	
*Alkalimonas amylolytica* N10	61	50	9.5	G1–G7	–	–	–	R	–	–	NR	R	R	R	R	NR	Wang et al. (2006)
				–	–	–	–	–	–	R	–	–	–	–	–	–	
*Anoxybacillus flavothermus*	70	65	7.5	R	–	–	–	–	–	–	–	–	–	–	–	–	Tawil et al. (2012)
				–	–	–	–	–	–	G1–G7	–	–	–	–	–	–	
*Anoxybacillus vranjensis* ST4	66	75	7	R	–	–	–	–	–	–	–	–	–	–	–	–	Slavić et al. (2023)
				–	G1–G7	–	–	G1–G6	–	G1–G7	–	–	–	–	–	–	
*Bacillus acidicola* TSAS1	62	60	4	G2–G5	–	–	–	–	R	R	R	R	R	–	–	NR	Sharma and Satyanarayana (2012)
				–	R	–	–	–	–	–	–	–	–	–	–	–	
*Bacillus amyloliquefaciens* ATCC 23842	58	50	5	G_O_	–	–	–	–	–	–	–	R	–	–	–	–	Alikhajeh et al. (2010; Gangadharan et al. (2010)
				–	R	R	–	R	R	R	–	R	–	–	–	–	
Degrade raw and gelatinized starches																	
*Bacillus paralicheniformis* ATCC9945a	55	90	6.5	R	–	–	–	–	–	–	–	–	–	–	–	–	Božić et al. (2011, (2020)
				–	R	–	–	R	–	R	–	–	–	–	–	–	
*Bacillus* sp. YX	58	45	5.5	G1, G2, G_O_	–	–	–	–	–	–	–	–	–	–	–	–	Liu and Xu (2008)
				–	R	–	–	R	–	R	–	–	–	–	–	–	
*Bacillus* sp. TS-23	69.5	65	8.5	R	–	–	–	–	–	–	–	–	–	–	–	–	Lin et al. (1994)
				–	–	–	–	–	–	G1–G5	–	–	–	–	–	–	
*Cytophaga* sp.	59	50	–	R	–	–	–	–	–	–	–	–	–	–	–	–	Jeang et al. (2002)
				–	–	–	–	–	–	R	–	–	–	–	–	–	
*Geobacillus thermoleovorans* NP54	59	80	5	G2, G3, G5	–	R	R	R	–	–	NR	R	R	–	R	NR	Mehta and Satyanarayana (2013)
				–	G2, G3, G5	–	–	–	–	G2, G3, G5	–	–	–	–	–	–	
*Halothermothrix orenii* H 168	57	65	8	R	–	–	–	–	–	–	NR	R	R	–	–	NR	Tan et al. (2008)
				–	–	–	–	–	–	R	–	–	–	–	–	–	
*Pontibacillus* sp. ZY	55	35	7	G1–G5	–	–	–	–	–	–	NR	R	R	–	–	NR	Fang et al. (2019)
				–	G2	–	–	G1–G5	G1–G5	G1–G5	–	–	–	–	–	–	
Degrade gelatinized starch																	
*Alicyclobacillus* sp. 18,711	54	–	–	R	–	–	–	–	–	–	R	–	–	–	–	–	Agirre et al. (2019)
*Bacillus ceres* GUF8	56	50	6	G2–G7	–	–	–	–	–	–	–	–	–	–	–	–	Mahdavi et al. (2010 )
*Bacillus licheniformis* ATCC 27811	52	70	8	R	–	–	–	–	–	–	–	–	–	–	–	–	Machius et al. (1998; Muazzam et al. (2019)
*Bacillus licheniformis* NH1	58	90	6.5	G2, G3, G5	–	–	–	–	–	–	–	–	–	–	–	–	Hmidet et al. (2008)
*Bacillus halmapalus* DSM8723	57	–	–	R	–	–	–	–	–	–	–	–	–	–	–	–	Lyhne-Iversen et al. (2006)
*Bacillus* sp. KSM-K38	55	55	8	G2, G3, G6, G7	–	–	–	R	–	R	NR	R	R	R	R	NR	Hagihara et al. (2001; Nonaka et al. (2003)
*Bacillus* sp. KSM-1378	53	55	8	G1–G7	–	–	–	–	–	–	NR	R	R	R	R	NR	Igarashi et al. (1998; Shirai et al. (2007)
*Bacillus* sp. MK-716	59	70	5.6	R	–	–	–	–	–	–	–	–	–	–	–	–	Sidhu et al. (1997)
*Exiguobacterium* sp. DAU5	57	40	8.5	G2, G3, G5	–	–	–	–	–	–	NR	–	–	–	–	–	Chang et al. (2013)
*Geobacillus stearothermophilus* DY5	59	105	6.5	R	–	–	–	–	–	–	–	–	–	–	–	–	Diderichsen et al. (1991); Suvd et al. (2001)
*Nostoc* sp. PCC 7119	56	31	7	G1–G8	–	–	–	–	–	–	NR	–	–	G1–G7	G6–G7	NR	Reyes-Sosa et al. (2010)
*Petrotoga mobilis*	56	80	7	R	–	–	–	R	R	R	NR	R	R	–	–	NR	Jabbour et al. (2013)
*Salmonella typhimurium* SJW1103	56	45	7.2	G2–G4	–	–	–	–	–	–	–	–	–	–	–	–	Raha et al. (1992)
*Streptococcus bovis* 148	57	40	6.5	Go	–	–	–	–	–	–	–	–	–	–	–	–	Satoh et al. (1997)
*Vibrio alginolyticus* 129–63	58	60	6	G1–G5	–	–	–	–	–	–	NR	R	R	R	–	NR	Liu et al. (2016)

Reaction products from gelatinized and raw starches are shown in black and red, respectively. *G1* glucose, *G2* maltose, *G3* maltotriose, *G4* maltotetraose, *G5*, maltopentaose, *G6* maltohexaose, *G7* maltoheptaose, *Go* oligosaccharides (≥ G8), *MM* molecular mass, *T*_Opt_ optimum temperature, *pH*_Opt_ optimum pH, *SS* soluble starch, *CS* corn starch, *PS* potato starch, *WS* wheat starch, *RS* rice starch, *TS* tapioca starch, *SA* sago starch, *P* pullulan, *AM* amylose, *AP* amylopectin, *D* dextrin, *G* glycogen, *β-CD* β-cyclodextrin, *RSS* raw soluble starch, *RCS* raw corn starch, *RPS* raw potato starch, *RWS* raw wheat starch, *RRC* raw rice starch, *RTS* raw tapioca starch, *RSA* raw sago starch, *RP* raw pullulan, *RAM* raw amylose, *RAP* raw amylopectin, *RD* raw dextrin, *RG* raw glycogen, *Rβ-CD* raw β-cyclodextrin, *Ref.* Reference, *R* reduced, *NR* not reduced, – not determined. ^a^Reaction products were determined using HPLC, thin-layer chromatography, high-performance anion-exchange chromatography, or gel permeation chromatography in the respective reference. ^T^Type strain

**Fig. 4:**
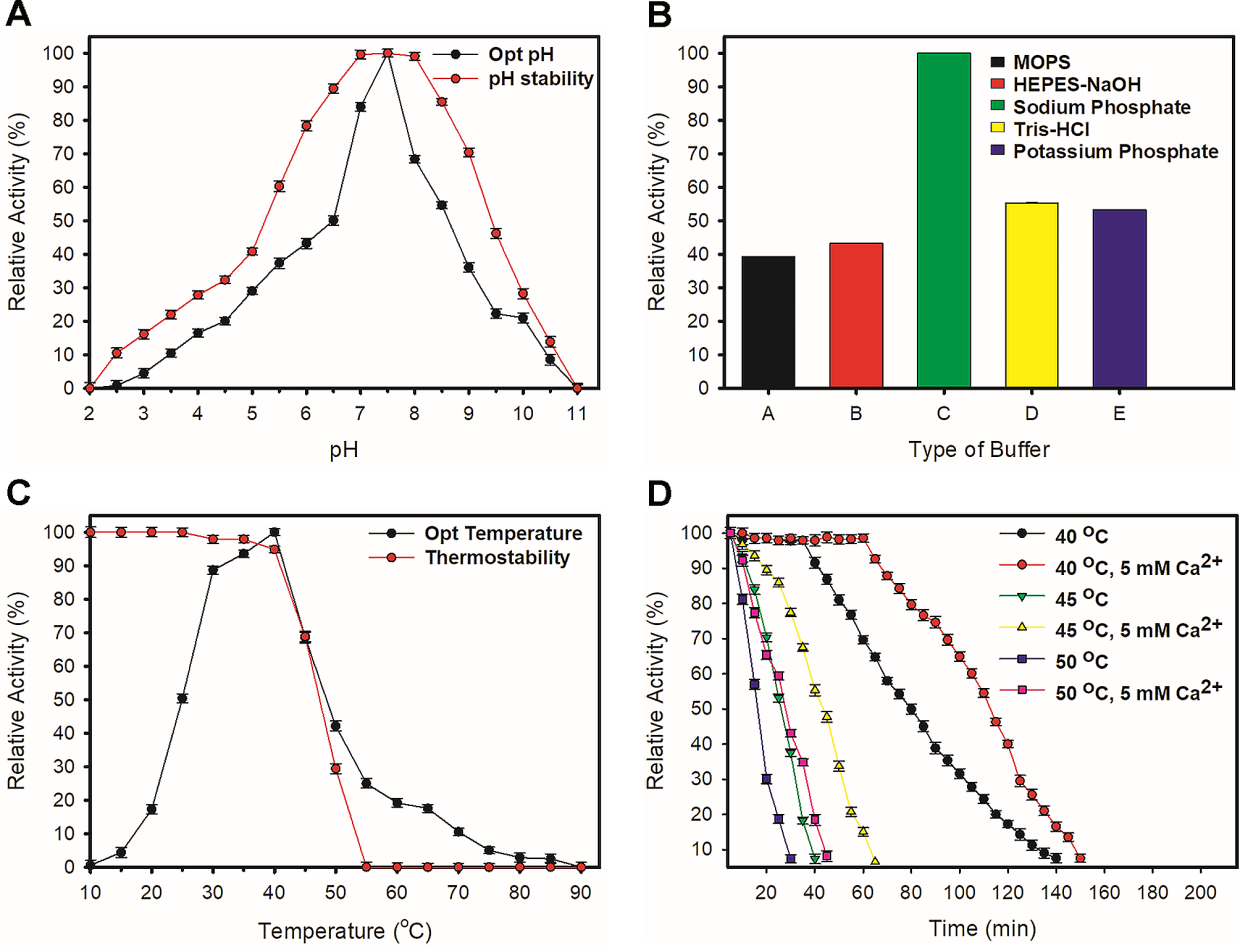
Biochemical characterization of AmyJM. **A** Effects of pH on AmyJM activity and stability. **B** Effects of buffer (100 mM each, pH 7.5) on AmyJM activity. **C** Effects of temperature on AmyJM activity and stability. **D** Thermostability of AmyJM at 40–50 °C (in the presence or absence of 5 mM calcium chloride). Data are shown as means ± standard errors of triplicate analyses

AmyJM exhibited optimum activity at pH 7.5 and was stable over a pH range of 5.5–9.0 (Fig. 4A). Among various buffers tested, 100 mM sodium phosphate buffer, pH 7.5 was the optimal buffer system for AmyJM catalytic activity (Fig. 4B). AmyJM was optimally active at 40 °C and remained stable between 10 and 45 °C after a 20-min incubation period (Fig. 4C). To further assess its thermostability, AmyJM was incubated at temperatures ranging from 40 to 50 °C for 2 h. AmyJM retained half-life activity after incubation at 40 °C for 80 min (Fig. 4D). The presence of 5 mM calcium chloride improved the thermostability of AmyJM. The enzyme remained stable (with > 50% activity) for up to 110 min at 40 °C (Fig. 4D). Based on an enzyme kinetics study (Additional file 1: Fig. S5), the *K*_m_ and *V*_max_ values of AmyJM were 8.84 mg/mL and 0.098 µmol/min/mg, respectively. The AmyJM *k*_cat_ and *k*_cat_/*K*_m_ values were 1.052 s^–1^ and 0.12 mg mL^–1^ s^–1^, respectively.

Table 2 summarizes the effects of various metal ions and chemical reagents on the catalytic activity of AmyJM. The addition of 5 mM calcium chloride increased AmyJM relative activity to 217.84%. As mentioned above, calcium chloride also positively influenced the thermostability of the enzyme (Fig. 4D). In contrast, other chloride metal ions (Co^2+^, Mn^2+^, and Fe^3+^) strongly inhibited AmyJM activity (> 80% activity loss). Among the chemical reagents tested (Table 2), AmyJM demonstrated a tolerance (retaining 99% of its activity) in the presence of Triton X-100 and Tween 20 at concentrations up to 5% (v/v). Other reagents (EDTA, urea, β-mercaptoethanol, and SDS) significantly reduced the AmyJM catalytic activity by more than 50%.

**Table 2:** Effects of various chloride metal ions and chemical reagents on AmyJM catalytic activity

Additives	Relative activity (%)	
	5 mM	10 mM
Chloride metal ions		
^a^Control	100.00 ± 0.02	100.00 ± 0.02
Ca^2+^	217.84 ± 0.04	130.27 ± 0.06
Mg^2+^	187.56 ± 0.07	112.53 ± 0.03
Na^+^	98.00 ± 0.02	60.10 ± 0.02
K^+^	97.20 ± 0.03	58.43 ± 0.07
NH_4_^+^	94.79 ± 0.05	53.87 ± 0.06
Zn^2+^	87.08 ± 0.03	42.24 ± 0.07
Cu^2+^	65.75 ± 0.04	39.45 ± 0.01
Ni^2+^	36.45 ± 0.07	21.87 ± 0.05
Co^2+^	22.91 ± 0.05	13.75 ± 0.03
Mn^2+^	17.07 ± 0.02	10.24 ± 0.02
Fe^3+^	11.67 ± 0.05	7.02 ± 0.01
Chemical reagents		
^a^Control	100.00 ± 0.02	100.00 ± 0.02
Ethylenediaminetetraacetic acid (EDTA)	46.19 ± 0.07	27.71 ± 0.01
Urea	38.58 ± 0.04	23.15 ± 0.07
β-mercaptoethanol	27.09 ± 0.01	17.73 ± 0.01

	5% (v/v)	10% (v/v)
^a^Control	100.00 ± 0.02	100.00 ± 0.02
Triton X-100	99.86 ± 0.01	59.92 ± 0.01
Tween 20	99.89 ± 0.01	58.73 ± 0.01
Tween 80	71.69 ± 0.01	43.01 ± 0.08
Dimethylsulfoxide (DMSO)	49.27 ± 0.09	36.16 ± 0.06
Sodium dodecyl sulfate (SDS)	36.83 ± 0.05	22.09 ± 0.03

^a^Enzyme activity in the absence of the additives was used as a reference (100%). Values are means ± standard errors from triplicate analyses

**Fig. 5:**
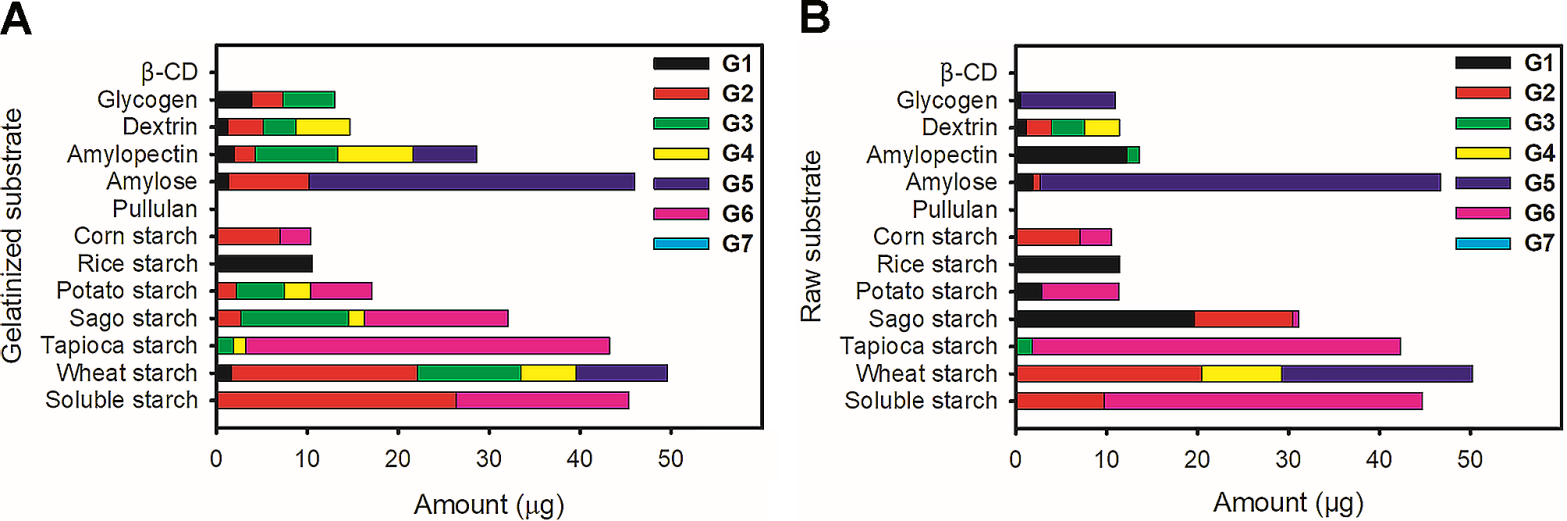
HPLC-ELSD analysis of reaction products of AmyJM with different **A** gelatinized substrates and **B** raw substrates. Data are shown as means ± standard errors of triplicate analyses. *G1* glucose, *G2* maltose, *G3* maltotriose, *G4* maltotetraose, *G5* maltopentaose, *G6* maltohexaose, *G7* maltoheptaose

The reaction product (sugar) profile of AmyJM on various gelatinized substrates was determined using HPLC-ELSD (Fig. 5A). The enzyme degraded a broad range of gelatinized starches, including soluble starch and wheat, tapioca, sago, potato, rice, and corn starches (1% w/v each). The products of starch hydrolysis by AmyJM were mixtures of glucose, maltose, maltotriose, maltotetraose, maltopentaose, and maltohexaose (G1–G6). Among the tested gelatinized starches, wheat starch was the best substrate for maximum total reducing sugar production by AmyJM (Fig. 5A).

### Raw starch degradation by AmyJM

The potential of AmyJM to hydrolyze raw starches was investigated by examining its enzyme adsorption and hydrolytic activities on various raw starch granules (1% w/v each) during a short 3-h incubation at 40 °C. The findings are summarized in Table 3. Adsorption studies indicated that AmyJM can bind to various raw starches. Among the raw starches tested, AmyJM exhibited the highest adsorption (53.20%) on raw wheat starch, followed by raw tapioca starch (35.10%), raw sago starch (30.10%), raw potato starch (20.30%), raw rice starch (20.20%), and raw corn starch (20.10%). AmyJM efficiently degraded all bound raw starches. Notably, the hydrolytic activity of AmyJM consistently correlated with its adsorption rate across the tested raw starches. Raw wheat starch, showing the highest adsorption, was the most susceptible to hydrolysis, with a degradation rate of 51.10%. This was followed by raw starches derived from tapioca, sago, potato, rice, and corn, which yielded 30.05%, 25.03%, 17.02%, 16.04%, and 16.01%, respectively.

**Table 3:** Adsorption and hydrolytic activities of AmyJM toward different raw starches

Starch characteristic					^a^AmyJM raw starch degradation performance	
Starch source	^b^Starch type (Granule crystalline type)	^c^Granule shape (Granule distribution)	^d^Amylose: amylopectin ratio (%)	^b^Granule size (Average size) (µm)	Adsorption (%)	Hydrolysis (%)
Wheat	Cereal (A-type)	Polyhedral (Bimodal)	24:76	5–10 (7.5)	53.20 ± 0.01	51.10 ± 0.02
Tapioca	Root (C-type)	Lenticular (Unimodal)	25:75	17–21 (19)	35.10 ± 0.02	30.05 ± 0.01
Sago	Cereal (C-type)	Lenticular (Unimodal)	26:74	30–40 (35)	30.10 ± 0.03	25.03 ± 0.04
Potato	Tuber (B-type)	Lenticular (Unimodal)	27:73	24–26 (25)	20.30 ± 0.03	17.02 ± 0.02
Rice	Cereal (A-type)	Polyhedral (Unimodal)	28:72	4–5 (4.5)	20.20 ± 0.10	16.04 ± 0.03
Corn	Cereal (A-type)	Polyhedral (Unimodal)	29:71	13–20 (15)	20.10 ± 0.04	16.01 ± 0.02

^a^The enzymatic degradation was performed using reaction mixture of AmyJM (40 U) with 1% (w/v) for each raw starch. The values are means ± standards error from triplicate analyses

^b^The starch characteristics were derived from previous studies (Chen et al. 2016; Mathobo et al. 2021)

^c^The shape, distribution, and diameter size of raw starch granules were assessed by FESEM

^d^The amylose/amylopectin ratio for each starch was determined using an Amylose/Amylopectin Assay Kit (Megazyme, County Wicklow, Ireland, UK).

**Fig. 6:**
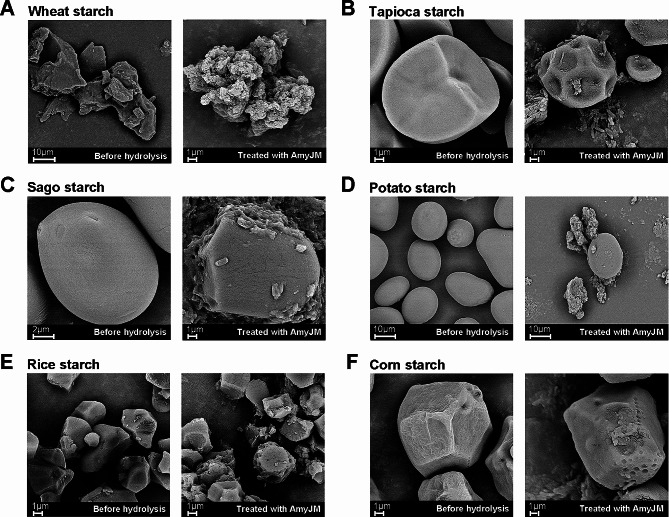
FESEM images of untreated and AmyJM-hydrolyzed raw starches. **A** Wheat starch. **B** Tapioca starch. **C** Sago starch. **D** Potato starch. **E** Rice starch. **F** Corn starch

FESEM was used to observe the changes in raw starch granules following digestion by AmyJM (Fig. 6). Initially, the untreated raw starch granules displayed intact structures with smooth surfaces; however, upon hydrolysis by AmyJM, they showed significant structural disruptions and unevenly distributed holes on their surfaces. The degree of granule alterations corresponded directly with the AmyJM degradation rates for the different raw starches (Table 3). AmyJM exhibited the highest hydrolytic activity (51.10%) toward raw wheat starch, resulting in extensive granule degradation characterized by an approximately 10-fold reduction in granule size and the formation of irregular shapes (Fig. 6A). In contrast, raw corn starch granules displayed only small holes on their surface, indicating the relatively lower activity (16.01%) of AmyJM toward this polysaccharide (Fig. 6F).

To investigate the reaction products, AmyJM was incubated with various 1% (w/v) raw substrates. The reaction products were analyzed using HPLC-ELSD (Fig. 5B). Similar to the products for gelatinized starches, AmyJM produced a mixture of reducing sugars (G1–G6) from various types of raw starches (Fig. 5). Among the raw starches tested, raw wheat starch was the best substrate for maximum total sugar production by AmyJM (Fig. 5B). Based on the combined data, the characteristics of AmyJM suggest its application potential for direct degradation of raw wheat starch in the food and fermentation industries.

## Discussion

*Jeotgalibacillus* is a seldom-explored genus of halophilic marine bacteria (Goh et al. 2019) with only one industrial GH enzyme previously documented (Liew et al. 2018). This study addressed the knowledge gap concerning GH enzymes within this genus, specifically investigating AmyJM. The strength of this study lies in the substantial relevance of α-amylases in numerous commercial applications, including starch liquefaction and saccharification processes, food and beverage production, and bioethanol generation (Farias et al. 2021; Farooq et al. 2021; Miao et al. 2018; Zhang et al. 2017, 2021). To date, no other α-amylase from this genus has been described.

Through primary sequence-based comparison, AmyJM exhibited all eight CSRs typically found in well-known bacterial α-amylases of the GH13_5 subfamily (Additional file 1: Figs. S1 and S2). In contrast to other subfamilies, AmyJM retains its unique CSRs feature, distinguishing the enzyme from other GH13 subfamilies (Additional file 1: Fig. S3). Based on its closer identity to representatives of the GH13_5 subfamily (Fig. 1) and divergence from α-amylase sequences in other subgroups, we classified this enzyme into the GH13_5 subfamily. Furthermore, the putative tertiary structure of AmyJM revealed a distinct length of domain B (Fig. 2), a characteristic identified in the crystal structures of GH13_5 α-amylases (Božić et al. 2020; Janeček et al. 2014; Lyhne-Iversen et al. 2006; Tan et al. 2008).

A comparative analysis of AmyJM and 27 well-studied GH13_5 bacterial α-amylases revealed various biochemical properties within the subfamily members. AmyJM showed optimal activity at 40 °C and pH 7.5, aligning with the optimal growth conditions for strain D5^T^ (Yaakop et al. 2015). The optimal temperature of AmyJM was similar to that of GH13_5 α-amylases from mesophiles (e.g., *Bacillus* and *Nostoc* spp.), exhibiting activity at moderate temperatures of 31–55 °C (Gangadharan et al. 2010; Liu and Xu 2008; Nonaka et al. 2003; Reyes-Sosa et al. 2010; Shirai et al. 2007). In contrast, thermophile enzymes (e.g., *Anoxybacillus* and *Geobacillus* spp.) have higher optimum temperatures, ranging from 65 to 105 °C (Diderichsen et al. 1991; Mehta and Satyanarayana 2013; Slavić et al. 2023; Tawil et al. 2012). Regarding the optimal pH, most GH13_5 α-amylases displayed optimal activity under acidic conditions, within the pH range of 4.0–6.9 (Table 1). Notable exceptions are found in *Nostoc* sp. PCC 7119, *Petrotoga mobilis*, and *Pontibacillus* sp. ZY, whose enzymes are most active at neutral pH (Fang et al. 2019; Jabbour et al. 2013; Reyes-Sosa et al. 2010). Several GH13_5 α-amylases, such as AmyJM and those from *(A) amylolytica* N10, *Bacillus* sp. TS-23, *(B) licheniformis* ATCC 27,811, and *Salmonella typhimurium* SJW1103 exhibit optimal activity in alkaline conditions (pH 7.2–9.5) (Lin et al. 1994; Muazzam et al. 2019; Raha et al. 1992; Wang et al. 2006).

The addition of calcium chloride improved the activity and thermostability of AmyJM; enzyme activity was increased by approximately 117%, and its half-life activity at 40 °C was extended by an additional 30 min (Fig. 4D). This positive influence of calcium chloride has been observed in other GH13_5 bacterial α-amylases, such as those from *Pontibacillus* sp. ZY, *P. mobilis*, and *A. vranjensis* ST4 (Fang et al. 2019; Jabbour et al. 2013; Slavić et al. 2023). In the homology model of AmyJM, a predicted calcium-binding site was identified to harbor a Ca^2+^ ion (Fig. 2). Subsequent comparison with the structures of nine GH13_5 bacterial α-amylases revealed the presence of this calcium-binding site in AmyJM, as well as its subfamily counterparts (Agirre et al. 2019; Alikhajeh et al. 2010; Božić et al. 2020; Lyhne-Iversen et al. 2006; Machius et al. 1998; Nonaka et al. 2003; Shirai et al. 2007; Suvd et al. 2001; Tan et al. 2008). This conserved binding pocket was located at the interface between domains A and B, which is in close proximity to the active site. Within this binding site, a pair of residues, commonly Asn and Asp, interact with either a single Ca^2+^ ion or a more complex metal ion arrangement, such as Ca^2+^-Na^+^-Ca^2+^, leading to structural stability in the enzymes. This stabilization mechanism is a key contributor to the observed enhancements in activity and thermostability within GH13_5 bacterial α-amylases (Božić et al. 2020; Machius et al. 1998; Tan et al. 2008).

The hydrolytic profile of AmyJM revealed significant activity against a range of gelatinized starches and related polysaccharides, resulting in the formation of reducing sugars (G1–G6) (Fig. 5). The broad substrate specificity of AmyJM and other GH13_5 bacterial α-amylases toward various gelatinized substrates (Table 1) is likely attributed to the presence of SBS. Previous studies have suggested that SBSs present in BHA, *Bli*Amy, and AmyB are essential for substrate adsorption and recognition (Božić et al. 2020; Lyhne-Iversen et al. 2006; Tan et al. 2008).

Enzymes capable of degrading raw starches (i.e., raw starch-degrading α-amylases) offer an alternative approach to sugar production through direct starch hydrolysis (Božić et al. 2017; Farias et al. 2021; Li et al. 2023; Zhang et al. 2017). AmyJM exhibits a pronounced preference for raw wheat starch compared with other tested raw starches. This preference may be attributed to the relatively lower amylose content of wheat starch (24%) compared with the other starch sources (27% on average) (Table 3). In general, starches with low amylose contents (e.g., wheat) have less compact granule structures, providing better accessibility and higher freedom for the enzyme to react with the starches, resulting in greater degradation efficiency, as supported by earlier reports (Božić et al. 2011; Chakraborty et al. 2020; Slavić et al. 2023; Tester et al. 2004; Wang et al. 2022). In relative terms, starches with high amylose contents (e.g., tapioca, sago, potato, rice, and corn) have densely packed granule structures, which limits enzyme access and makes them less prone to enzymatic action (Bertoft 2017; Chakraborty et al. 2020; Wang et al. 2022).

The ability of AmyJM to degrade raw starch may be attributed to the presence of the residue pair Y259 and Y360, which correspond to the putative SBS in AmyJM (analogous to *Bli*Amy SBS F257 and Y358). Previous research has emphasized the role of SBS from *Bli*Amy in the binding and degradation of raw starches (Božić et al. 2020); using *Bli*Amy mutants, the authors demonstrated the importance of SBS in recognizing and adsorbing raw starch granules. By comparing the SBS of *Bli*Amy (residues F257 and Y358) with those of AmyJM (residues Y259 and Y360), it was observed that the former Tyr residue replaced the Phe in *Bli*Amy. As both Tyr and Phe are amino acids with aromatic rings, this substitution is unlikely to significantly affect the interactions with raw starch granules at this site. Moreover, aromatic residues (Tyr, Phe, and Trp) are known to be involved in stacking interactions of GH13 α-amylases with gelatinized substrates, raw substrates, and oligosaccharides (Božić et al. 2017; Kumar 2010; Miao et al. 2018). Therefore, we anticipate that the pair of residues Y259 and Y360 in AmyJM serve a similar function as their SBS F257 and Y358 counterparts in *Bli*Amy, which facilitate the binding and degradation of raw starches. Future experiments with SBS mutants will provide insights into the contributions of these residues to the efficiency of raw starch adsorption and hydrolysis by AmyJM.

In summary, this study successfully expressed, purified, and biochemically characterized a recombinant α-amylase from *J. malaysiensis* D5^T^ (designated as AmyJM). The enzyme was categorized within the GH13_5 subfamily of α-amylases. AmyJM demonstrated advantages in direct raw starch saccharification owing to its ability to hydrolyze raw wheat starch at low temperatures effectively.

### Electronic supplementary material

Below is the link to the electronic supplementary material.

Fig. S1 Nucleotide and amino acid sequences of recombinant AmyJM. Fig. S2 Multiple sequence alignment of AmyJM and all 27 well-characterized bacterial α-amylases from subfamily GH13_5 using Clustal Omega. Fig. S3 A Comparison of conserved sequence regions (CSRs) between AmyJM and representatives of bacterial α-amylases from the GH13 family using WebLogo3. B Comparison of CSRs between AmyJM and all 27 well-characterized bacterial α-amylases belonging to subfamily GH13_5 using WebLogo3. Fig. S4 Multiple sequence alignment of BliAmy, AmyB, and BHA surface-binding sites (SBSs) with AmyJM using Clustal Omega. Fig. S5 Rate vs. substrate concentration plot for AmyJM hydrolysis of soluble starch at different concentrations (2‒40 mg/mL). Supplementary Material 1

## Data Availability

The data for complete genome sequence of *J. malaysiensis* D5^T^ are publicly available in NCBI under BioProject accession number PRJNA253510, BioSample accession number SAMN02870886, and GenBank accession number CP009416.1. The 16S rRNA gene sequence of *J. malaysiensis* D5^T^ was deposited in NCBI GenBank under accession number KJ460028. The locus-tag of *amyJM* gene sequence and AmyJM protein sequence were deposited in the NCBI GenBank under the accession numbers of JMA_33490 and AJD92666.1, respectively. The AmyJM protein entry is publicly available in the UniProtKB database under accession number A0A0B5ARF3. The homology model of AmyJM is publicly available in the AlphaFoldDB protein structure database under model number AF-A0A0B5ARF3-F1.

## Abbreviations

β-CD: β-cylcodextrin
AmyB: α-amylase from *Halothermothrix orenii* H 168
AmyJM: α-amylase from *Jeotgalibacillus malaysiensis* D5^T^
BHA: α-amylase from *Bacillus halmapalus* DSM8723
*Bli*Amy: α-amylase from *Bacillus paralicheniformis* ATCC9945a
CAZy: Carbohydrate-Active enZYmes
CBM: Carbohydrate-binding module
CSR: Conserved sequence region
DNS: 3,5-dinitrosalicyclic acid
FESEM: Field emission scanning electron microscopy
GH: Glycoside hydrolase
GH13_5: Glycoside hydrolase family 13 subfamily 5
HPLC-ELSD: High-performance liquid chromatography with evaporative light scattering detection
LPSN: List of Prokaryotic Names with Standing in Nomenclature
MEGA: Molecular Evolutionary Genetic Analysis
MWCO: Molecular weight cut-off
SAVES: Structural Analysis and Verification Server
SBS: Surface-binding site
SDS-PAGE: Sodium dodecyl sulfate-polyacrylamide gel electrophoresis
