# Supplementary material for: Exploring a novel GH13_5 α-amylase from Jeotgalibacillus malaysiensis D5T for raw starch hydrolysis
Source: AMB Express. 2024 Jun 14;14:71. doi: 10.1186/s13568-024-01722-3 (PMC11178733; doi:10.1186/s13568-024-01722-3)
Supplement: Supplementary file 1 — Fig. S1 Nucleotide and amino acid sequences of recombinant AmyJM. Fig. S2 Multiple sequence alignment of AmyJM and all 27 well-characterized bacterial α-amylases from subfamily GH13_5 using Clustal Omega. Fig. S3 A Comparison of conserved sequence regions (CSRs) between AmyJM and representatives of bacterial α-amylases from the GH13 family using WebLogo3. B Comparison of CSRs between AmyJM and all 27 well-characterized bacterial α-amylases belonging to subfamily GH13_5 using WebLogo3. Fig. S4 Multiple sequence alignment of BliAmy, AmyB, and BHA surface-binding sites (SBSs) with AmyJM using Clustal Omega. Fig. S5 Rate vs. substrate concentration plot for AmyJM hydrolysis of soluble starch at different concentrations (2‒40 mg/mL). Supplementary Material 1 [file 13568_2024_1722_MOESM1_ESM.docx]

**Supplementary Information (Additional File 1)**

**Exploring a novel GH13_5 α-amylase from *Jeotgalibacillus malaysiensis* D5^T^ for raw starch hydrolysis**

Nurfatini Radzlin^1,2,3^, Mohd Shukuri Mohamad Ali^2,3,4^, Kian Mau Goh^5^, Amira Suriaty Yaakop^6^, Iffah Izzati Zakaria^1*^ and Ummirul Mukminin Kahar^1*^

^1^Malaysia Genome and Vaccine Institute, National Institutes of Biotechnology Malaysia, Jalan Bangi, 43000 Kajang, Selangor, Malaysia

^2^Enzyme and Microbial Technology Research Centre, Faculty of Biotechnology and Biomolecular Sciences, Universiti Putra Malaysia, 43400 Serdang, Malaysia

^3^Department of Biochemistry, Faculty of Biotechnology and Biomolecular Sciences, Universiti Putra Malaysia, 43400 Serdang, Selangor, Malaysia

^4^Enzyme Technology Laboratory, Institute Bioscience, Universiti Putra Malaysia, 43400 Serdang, Malaysia

^5^Department of Biosciences, Faculty of Science, Universiti Teknologi Malaysia, 81310 Skudai, Johor, Malaysia

^6^School of Biological Sciences, Universiti Sains Malaysia, 11800 Minden, Pulau Pinang, Malaysia

*Correspondences:

Ummirul Mukminin Kahar and Iffah Izzati Zakaria

ummirul@nibm.my and iffahizzati@nibm.my

| 1  1  61  21  121  41  181  61  241  81  301  101  361  121  421  141  481  161  541  181  601  201  661  221  721  241  781  261  841  281  901  301  961  321  1021  341  1081  361  1141  381  1201  401  1261  421  1321  441  1381  461  1441  481 | ttaagaaggagatataccatgggcagcagccatcatcatcatcatcacagcagcggcctg  **Thrombin**  **site**  **His tag**  H H H H H H S S G L  ***Bam*HI**  **T7 tag**  ***Nde*I**  **Thrombin site**  ***Nhe*I**  gtgccgcgcggcagccatatggctagcatgactggtggacagcaaatgggtcgcggatcc  V P R G S H M A S M T G G E E M G R G S  gtggaaagaaatcatacaatgcttcaattcttcgagtggcacgtcgctgcagacggtgac  M E R N H T M L Q F F E W H V A A D G D  **CSR VIII**  cactggaaacgactaaaggatgccgcacccgaactgaaagccagcggaattgacgcagtc  H W K R L K D A A P E L K A S G I D A V  **CSR VI**  tggattcctcctgttacaaaaggtgaatcacctgatgataacggctatggcgtatacgac  W I P P V T K G E S P D D N G Y G V Y D  **CSR VI (cont.)**  ctttatgaccttggagaattcgatcaaaaaggtgacgtcagaaccaaatacggaacgaaa  L Y D L G E F D Q K G D V R T K Y G T K  gacgagcttcatgaagccatccaggcctgccatgaaaacgacattcaggtatacgtagat  D E L H E A I Q A C H E N D I Q V Y V D  **CSR I**  gtggtcatgaaccataaagcaggcgcagatgaaaaagaaacgtttcaggtcattgaagta  V V M N H K A G A D E K E T F Q V I E V  **CSR I (cont.)**  gacgctgaaaaccgccttgaagagatctctgaaccatttgagattgaaggctggacgaag  D A E N R L E E I S E P F E I E G W T K  tttactttcccaggacgtgatggcaagtactccgatttccagtggaactttgaccatttc  F T F P G R D G K Y S D F Q W N F D H F  aacggcacagactatgatgcccgtgaagaaagaacaggctttttcaaaattgtcggtgag  N G T D Y D A R E E R T G F F K I V G E  ggaaaagactggaatgatcaggtcgacgatgagtttggaaattatgattacctcatgttt  G K D W N D Q V D D E F G N Y D Y L M F  **CSR V**  gcaaacattgattataatcaccctgaagtccgtgatgagatgatcaattggggtaaatgg  A N I D Y N H P E V R D E M I N W G K W  **CSR V (cont.)**  atgtcagatacacttgatgcagacggctaccgccttgatgcaatcaaacatatcagccat  M S D T L D A D G Y R L **D** A I K H I S H  **CSR II**  gaatttatcaatgaatttatcagacagataaaagagcatcgtgatggcaatttttatatg  E F I N E F I R Q I K E H R D G N F Y M  **CSR III**  gtcggcgaatactggaatgaagatattgaagcctgcggaaacttccttgaggcgacggat  V G **E** Y W N E D I E A C G N F L E A T D  **CSR III (cont.)**  tatcagcttgatttattcgatgttcagctgcattataagcttcaccgcatttcagaagaa  Y Q L D L F D V Q L H Y K L H R I S E E  ggccgtgatgcagacctttcaaccctttttgaagggacacttgtcgaagaattcccgatg  G R D A D L S T L F E G T L V E E F P M  aatgctgtaacgtttgttgataaccatgattctcagcctggtgagtcacttgaatcgtgg  N A V T F V D N H **D** S Q P G E S L E S W  **CSR IV**  gtacaggattggtttaagcagagtgcctatgcgcttgtgctgcttcgtgcagatggctac  V Q D W F K Q S A Y A L V L L R A D G Y  **CSR VII**  ccttgcgtattttacggggattacttcggtattgatggagaagatccgattgaggataaa  P C V F Y G D Y F G I D G E D P I E D K  **CSR VII (cont.)**  I  cgtgaagcgattgatccgctgatttacgcgcgctatcatcgtgcttatggtgagcaggac  R E A I D P L I Y A R Y H R A Y G E Q D  gattatttcgatcatcctgccacgattggctgggtcagacgcggtgttgaagaaattgaa  D Y F D H P A T I G W V R R G V E E I E  ggttcaggctgtgcagtcgttgtcacagttggtgatgagggtgaaaagagaatgtttgtt  G S G C A V V V T V G D E G E K R M F V  ggtgaacatcgcgcaggcgaagaatggacggattacacgaattcgattgatgagcctgtt  G E H R A G E E W T D Y T N S I D E P V  gtgattgatgatgaaggatatgggactttcccagttcaggcgggcagtgtgtcggtttgg  V I D D E G Y G T F P V Q A G S V S V W  **His tag**  ***Xho*I**  gctttgccggctgaactcgagcaccaccaccaccaccactga  A L P A E L E H H H H H H **Stop** |
| --- | --- |

**Fig. S1** Nucleotide and amino acid sequences of recombinant AmyJM. Residues M1–Y396; E209–Y396 represent the catalytic domain (Domain A), highlighted in blue. Residues A109–P208 represent the extended loop domain (Domain B), highlighted in cyan. Residues G397–E485 represent the C-terminal domain (Domain C), highlighted in red. D233, E263, and D330 represent the catalytic triad, shown in orange. The eight conserved regions of α- amylase, designated as CSRs I, II, III, IV, V, VI, VII, and VIII, are indicated by rectangle boxes. Putative calcium-binding sites (N104 and D196) are shown in brown. Putative surface-binding site, SBS (Y259 and Y360) are shown in gray. Histidine tags (His tags) at the N- and C-termini are highlighted in green. The full-length AmyJM construct, including the AmJM mature sequence, His tags at both N- and C-termini, and other additional residues within the pET-28a(+) plasmid, had a theoretical molecular weight of 60.11 kDa.

AmyJM ------------------------------------------------------------ 0

BHA ------------------------------------------------------------ 0

BliAmy ------------------------------------------------------------ 0

AmyB GCSNISEDVNNPNRSLFLIESEPSTGASVSKNLTEIILIFSNDINKVSQLALTDLITDSD 60

AmyA ------------------------------------------------------------ 0

AmyII ------------------------------------------------------------ 0

Amy1 ------------------------------------------------------------ 0

Amy63 ------------------------------------------------------------ 0

AmyK38 ------------------------------------------------------------ 0

AliC ------------------------------------------------------------ 0

Amy ------------------------------------------------------------ 0

AVA ------------------------------------------------------------ 0

AFA ------------------------------------------------------------ 0

AmyS ------------------------------------------------------------ 0

AmyMK-716 ------------------------------------------------------------ 0

AmyTSAS1 ------------------------------------------------------------ 0

Gt-Amy ------------------------------------------------------------ 0

LAMY ------------------------------------------------------------ 0

AmyH ------------------------------------------------------------ 0

AmyZ1 ------------------------------------------------------------ 0

BCA ------------------------------------------------------------ 0

AmyC ------------------------------------------------------------ 0

BAA ------------------------------------------------------------ 0

BYXA ------------------------------------------------------------ 0

BLA ------------------------------------------------------------ 0

AmyN ------------------------------------------------------------ 0

AmyBN10 ------------------------------------------------------------ 0

Amy13A ------------------------------------------------------------ 0

AmyJM ------------------------------------------------------------ 0

BHA ------------------------------------------------------------ 0

BliAmy ------------------------------------------------------------ 0

AmyB IQGIDYNIEGNKVIINNFSLEPTCN----YRLSYEVIDIYDNHLQGYIEFLVNQSNYPQI 116

AmyA ------------------------------------------------------------ 0

AmyII ------------------------------------------------------------ 0

Amy1 ------------------------------------------------------------ 0

Amy63 -------------------MKTF-------------N----LKPTLLPLTLL----LSSP 20

AmyK38 ------------------------------------------------------------ 0

AliC ------------------------------------------------------------ 0

Amy -----------------------M------NNVKKVW-LYYSIIATLVISFFTPFSTAQA 30

AVA -----------------------M------NYLKKVW-LYYAIVATLIISFLTPFSTAQA 30

AFA ------------------------------------------------------------ 0

AmyS ------------------------------------------------------------ 0

AmyMK-716 -------------------MLTFH------RIIRKGW-MFLL-AFLLTASLFCPTGQPAK 33

AmyTSAS1 ------------------------------------------------------------ 0

Gt-Amy -------------------MLTFH------RIIRKGW-MFLL-AFLLTASLFCPTGQPAK 33

LAMY ------------------------------------------------------------ 0

AmyH -------------------MLKKR------QGI---AVLAGVTSIAL---LSG--QPVAQ 27

AmyZ1 --------------------------------------MLGISF-VFIVSMFL--PTSHV 19

BCA -------------------------------MFKRIT-IVGLSVVMFLPSIYE--GSKAY 26

AmyC -------------------MKKKW------KKRSRFAAITMFSTMLLVPSLAQ--P-KEA 32

BAA ------------------------------------------------------------ 0

BYXA -------------------MIQKR------KRTVSFRLVLM-CTLLF----VS--L-PIT 27

BLA ------------------------------------------------------------ 0

AmyN -------------------MKQHK------RLYARLLPLLF-ALIFL----LS--H-SAA 27

AmyBN10 -------------------MQKTAKTSIWQRMRHSAIAL----SALSLSFGLQASELPQI 37

Amy13A ----------------------------------------------------MKDNFPSV 8

AmyJM --MERNHTMLQFFEWHVAADG---------DHWKRLKDAAPELKASGIDAVWIPPVTKGE 49

BHA HHNGTNGTMMQYFEWHLPNDG---------NHWNRLRDDASNLRNRGITAIWIPPAWKGT 51

BliAmy --ASLNGTLMQYFEWYMPNDG---------QHWKRLQNDSAYLAEHGITAVWIPPAYKGT 49

AmyB PDQEVNHTILQAFYWEMNTGEYATEHPEEANLWNLLAERAPELAEAGFTAVWLPPANKGM 176

AmyA ---MKNPTLLQYFHWYYPDGG---------KLWSELAERADGLNDIGINMVWLPPACKGA 48

AmyII ----TNETLMQYFEWYLPNDG---------KHWMRLAADAPTLAQKGITKIWMPPAFKAT 47

Amy1 ----MNGTMMQYFHWYIPNDG---------NLWSKVEASAPELADAGFTAMWLPPAYKGF 47

Amy63 VMAAQNGTMMQYFHWYVPNDG---------ALWTQVENNASALSDNGFTALWLPPAYKGA 71

AmyK38 --DGLNGTMMQYYEWHLENDG---------QHWNRLHDDAAALSDAGITAIWIPPAYKGN 49

AliC TFAGDNGTMMQYFEWYLPNDG---------TLWTKMGSDASHLKSIGITGVWFPPAYKGQ 51

Amy NTAPINETMMQYFEWDLPNDG---------TLWTKVKNEAANLSSLGITALWLPPAYKGT 81

AVA NTAPVNGTMMQYFEWDLPNDG---------TLWTKVKNEAANLSSLGITALWLPPAYKGT 81

AFA GSVPVNGTMMQYFEWYLPDDG---------TLWTKVANNAQSLANLGITALWLPPAYKGT 51

AmyS -AAPFNGTMMQYFEWYLPDDG---------TLWTKVANEANNLSSLGITALWLPPAYKGT 50

AmyMK-716 AAAPFNGTMMQYFEWYLPDDG---------TLWTKVANEANNLSSLGITALWLPPAYKGT 84

AmyTSAS1 ----FNGTMMQYFEWYLPDDG---------TLWTKVANEANNLSSLGITALWLPPAYKGT 47

Gt-Amy AAAPFNGTMMQYFEWYLPDDG---------TLWTKVANEANNLSSLGITALWLPPAYKGT 84

LAMY HHNGTNGTMMQYFEWHLPNDG---------NHWNRLRDDAANLKSKGITAVWIPPAWKGT 51

AmyH AATPQNGTMMQYFEWYVPNDG---------LHWNHLSNDSQHLKDIGISTVWIPPAYKGT 78

AmyZ1 NAASKNGTMMQYFEWYLPNSG---------THWNNLKNDAGHLNDLGITAVWIPPAYKGT 70

BCA ADTVNNGTLMQYFEWYAPNDG---------NHWNRLRTDAENLAEKGITSVWIPPAYKGT 77

AmyC KAAATNGTMMQYFEWYVPNDG---------QQWNRLRTDAPYLSSVGITAVWTPPAYKGT 83

BAA ----VNGTLMQYFEWYTPNDG---------QHWKRLQNDAEHLSDIGITAVWIPPAYKGL 47

BYXA KTSAVNGTLMQYFEWYTPNDG---------QHWKRLQNDAEHLSDIGITAVWIPPAYKGT 78

BLA --ANLNGTLMQYFEWYMPNDG---------QHWKRLQNDSAYLAEHGITAVWIPPAYKGT 49

AmyN AAASLNGTLMQYFEWYMPNDG---------QHWKRLQNDSAYLAEHGITAVWIPPAYKGT 78

AmyBN10 PPQQINNTMYQAFYWDAYP-----------GLWANLPAMAAPLAERGITSMWLPPAAKGM 86

Amy13A S--RNNETILQAFYWEMGETG---HPKEEENLWLLLTQKAPELSEVGFTGVWLPPANKAM 63

**CSR VIII**

**CSR VI**

AmyJM S-PDDNGYGVYDLYDLGEFDQKGDVRTKYGTKDELHEAIQACHENDIQVYVDVVMNHKAG 108

BHA S-QNDVGYGAYDLYDLGEFNQKGTVRTKYGTRSQLESAIHALKNNGVQVYGDVVMNHKGG 110

BliAmy S-QDDVGYGAYDLYDLGEFHQKGTVRTKYGTKGELQSAINSLHSRDINVYGDVVINHKGG 108

AmyB AGIHDVGYGTYDLWDLGEFDQKGTVRTKYGTKGELENAIDALHNNDIKVYFDAVLNHRMG 236

AmyA SGGYSVGYDTYDLFDLGEFDQKGTIATKYGDKRQLLTAIDALKKNNIAVLLDVVVNHKMG 108

AmyII H-DGDVGYGVYDLFDLGEFDQKGTIRTKYGTKADYLQAISALKENHIAPLADVVLNHKAA 106

Amy1 AGSFDVGYGVYDLFDLGEFDQKGSVRTKYGTRQQYLDAVKSLQTHGLQVYADAVLNHKMG 107

Amy63 GGSNDVGYGVYDMYDLGEFDQQGSVRTKYGTKDQYLSAINTAHKNNIQIYGDVVFNHRGG 131

AmyK38 S-QADVGYGAYDLYDLGEFNQKGTVRTKYGTKAQLERAIGSLKSNDINVYGDVVMNHKMG 108

AliC S-QSDVGYGVYDMYDLGEFNQKGTVRTKYGTKAQLQSAITSLHNNGIQAYGDVVLNHRMG 110

Amy S-QSDVGYGVYDLYDLGEFNQKGTIRTKYGTKTQYIQAIQAAKAAGMQVYADVVFNHKAG 140

AVA S-QSDVGYGVYDLYDLGEFNQKGTIRTKYGTKTQYIQAIQAAKAAGMQVYADVVFNHKAG 140

AFA S-SSDVGYGVYDLYDLGEFNQKGTVRTKYGTKTQYIQAIQAAHTAGMQVYADVVFNHKAG 110

AmyS S-RSDVGYGVYDLYDLGEFNQKGAVRTKYGTKAQYLQAIQAAHAAGMQVYADVVFDHKGG 109

AmyMK-716 S-RSDVGYGVYDLYDLGEFNQKGAVRTKYGTKAQYLQAIQAAHAAGMQVYADVVFDHKGG 143

AmyTSAS1 S-RSDVGYGVYDLYDLGEFNQKGTVRTKYGTKAQYLQTIQAAHAAGMQVYADVVFDHKGG 106

Gt-Amy S-RSDVGYGVYDLYDLGEFNQKGTVRTKYGTKAQYLQAIQAAHAAGMQVYADVVFDHKGG 143

LAMY S-QNDVGYGAYDLYDLGEFNQKGTVRTKYGTRSQLQGAVTSLKNNGIQVYGDVVMNHKGG 110

AmyH S-QNDVGYGAYDLYDLGEFNQKGTTRTKYGTKAQLQSAISNLRGKGIGVYGDVVMNHKGG 137

AmyZ1 S-QDDVGYGAYDLYDLGEFNQKGTVRTKYGTKAQLKNAIQTLQQQGIQVYGDVVMNHKGG 129

BCA T-QNDVGYGAYDLYDLGEFNQKGTVRTKYGTKAQLKSAIDALHKKNIDVYGDVVMNHKGG 136

AmyC S-QADVGYGPYDLYDLGEFNQKGTVRTKYGTKGELKSAVNTLHSNGIQVYGDVVMNHKAG 142

BAA S-QSDNGYGPYDLYDLGEFQQKGTVRTKYGTKSELQDAIGSLHSRNVQVYGDVVLNHKAG 106

BYXA S-QSDNGYGPYDLYDLGEFQQKGTVRTKYGTKSELQDAIGSLHSRNVQVYGDVVLNHKAG 137

BLA S-QADVGYGAYDLYDLGEFHQKGTVRTKYGTKGELQSAIKSLHSRDINVYGDVVINHKGG 108

AmyN S-QDDVGYGAYDLYDLGEFHQKGTVRTKYGTKGELQSAINSLHSRDINVYGDVVINHKGG 137

AmyBN10 NGTFSVGYDVYDFWDLGEFYQKGTTATRYGSRQQLQQALAALDQLGIQAYFDVVFNHRMG 146

Amy13A GGKKDVGYATYDLWDLGEFNQKDTVRTKYGTKEELESAIKILHGNGIKVYYDAVLNHRMG 123

**CSR I**

AmyJM ADEKETFQVIEVDAENRLEEISEPFEIEGWTKFTFPGRDGKYSD---------FQWNFDH 159

BHA ADATENVLAVEVNPNNRNQEISGDYTIEAWTKFDFPGRGNTYSD---------FKWRWYH 161

BliAmy ADATEYVTAVEVDPADRNRVTSGEQRIKAWTHFQFPGRGSTYSD---------FKWYWYH 159

AmyB ADYAETVLLDENSRDK------PGQYIKAWTGFNFPGRNGEYSN---------FTWNGQC 281

AmyA ADEKERIRVQRVNQDDRTQIDDNIIECEGWTRYTFPARAGQYSN---------FIWDYHC 159

AmyII ADHTETFSVVEVAPDDRTKIISKPFDIEGWTNFTFDGRHKAYND---------FEWHWYH 157

Amy1 GDAVETPKATPFPQDDRLNPKGGLQDIKTYTHYNFPGRQGKYSN---------FEWHWWH 158

Amy63 ADGKSWVDTKRVDWNNRNIEL-GDKWIEAWVEFDFPGRNDKYSN---------FHWTWYH 181

AmyK38 ADFTEAVQAVQVNPTNRWQDISGAYTIDAWTGFDFSGRNNAYSD---------FKWRWFH 159

AliC ADATETISAVEVNPSNRNQVTSGAYNISAWTDFEFPGRGNTYSS---------FKWHSYY 161

Amy ADGTEFVDAVEVDPSNRNQETSGTYQIQAWTKFDFPGRGNTYSS---------FKWRWYH 191

AVA ADATEWVDAVEVNPSNRNQEVSGTYQIQAWTKFDFPGRGNTYSS---------FKWRWYH 191

AFA ADGTELVDAVEVNPSDRNQEISGTYQIQAWTKFDFPGRGNTYSS---------FKWRWYH 161

AmyS ADGTEWVDAVEVNPSDRNQEISGTYQIQAWTKFDFPGRGNTYSS---------FKWRWYH 160

AmyMK-716 ADGTEWVDAVEVNPSDRNQEISGTYQIQAWTKFDFPGRGNTYSS---------FKWRWYH 194

AmyTSAS1 ADGTEWVDAVEVNPSDRNQEISGTYQIQAWTKFDFPGRGNTYSS---------FKWRWYH 157

Gt-Amy ADGTEWVDAVEVNPSDRNQEISGTYQIQAWTKFDFPGRGNTYSS---------FKWRWYH 194

LAMY ADGTEMVNAVEVNRSNRNQEISGEYTIEAWTKFDFPGRGNTHSN---------FKWRWYH 161

AmyH ADYTESVQAVEVNPSNRNQETSGEYSISAWTGFNFAGRNNTYSP---------FKWRWYH 188

AmyZ1 ADFTEQVTAVEVNSGNRNVQTSGAYTIEAWTGFNFPGRGDAYSS---------FDWHWYH 180

BCA ADYTETVTAVEVDPSNRNIEVSGDYEISAWTGFNFPGRGDPYSN---------FKWKWYH 187

AmyC ADYTENVTAVEVNPSNRNQETSGEYNIQAWTGFNFPGRGTTYSN---------FKWQWFH 193

BAA ADATEDVTAVEVNPANRNQETSEEYQIKAWTDFRFPGRGNTYSD---------FKWHWYH 157

BYXA ADATEDVTAVEVNPANRNQETSGEYQIKAWTDFRSPGRGNTYSD---------FKWHWYH 188

BLA ADATEDVTAVEVDPADRNRVISGEHLIKAWTHFHFPGRGSTYSD---------FKWHWYH 159

AmyN ADATEDVTAVEVDPADRNRVTSGEQRIKAWTHFQFPGRGSTYSD---------FKWYWYH 188

AmyBN10 ADAQEHI----------------PGFGLAWTEYHLQGRQAHYTQQNWGYLWHDFDWDWTA 190

Amy13A ADSTETVKLSLNSPDK------PGQTILAWTKFTFPGRKNKYSD---------FQWNWQC 168

AmyJM FNGTDYDAREER-TGFFKIVGEGKDWND--QVDDEFGNYDYLMFANIDYNHPEVRDEMIN 216

BHA FDGVDWDQSRQFQNRIYKFRGDGKAWDW--EVDSENGNYDYLMYADVDMDHPEVVNELRR 219

BliAmy FDGTDWDESRKL-NRIYKFQ--GKAWDW--EVSNENGNYDYLMYADIDYDHPDVTAEIKR 214

AmyB FDGTDWDDYSKE-SGKYLF--DEKSWDW-----TYNWDEDYLMGADVDYENEAVQNDVID 333

AmyA FSGIDHIENPDE-DGIFKIVNDYTGDGWNDQVDDEMGNFDYLMGENIDFRNHAVTEEIKY 218

AmyII FTGTDYDVKTGK-NGIFQIQGDNKGWANQDLVDGENGNYDYLMYADLDFKHPEVIKNIYD 216

Amy1 FDAVDYNEYNSG-DRSTVYLLEGKNFDD--YVALEKGNFAYLMGCDLDFQNEWVRGEVTY 215

Amy63 FDGVDWDDAGEE-KAIFKFKGEGKAWDW--EVSSEKGNYDYLMYADLDMDHPEVKQELKD 238

AmyK38 FNGVDWDQRYQE-NHIFRFA--NTNWNW--RVDEENGNYDYLLGSNIDFSHPEVQDELKD 214

AliC FDGVDWDQSRQLSGKIYQIQ--GKAWDW--EVDSENGNYDYLMGADIDYDHPDVQTEVKN 217

Amy FDGTDWDESRKL-NRIYKFRSTGKAWDW--EVDTENGNYDYLMFADLDMDHPEVVTELKN 248

AVA FDGTDWDQSRQL-NRIYKFRGTGKAWDW--EVDTENGNYDYLMFADLDMDHPEVVAELKN 248

AFA FDGTDWDESRKL-NRIYKFRGTGKAWDW--EVDTENGNYDYLMYADLDMDHPEVVSELKN 218

AmyS FDGVDWDESRKL-SRIYKFRGIGKAWDW--EVDTENGNYDYLMYADLDMDHPEVVTELKS 217

AmyMK-716 FDGVDWDESRKL-SRIYKFRGIGKAWDW--EVDTENGNYDYLMYADLDMDHPEVVTELKN 251

AmyTSAS1 FDGVDWDESRKL-SRIYKFRGIGKAWDW--EVDTENGNYDYLMYADLDMDHPEVVTELKN 214

Gt-Amy FDGVDWDESRKL-SRIYKFRGIGKAWDW--EVDTENGNYDYLMYADLDMDHPEVVTELKN 251

LAMY FDGTDWDQSRQLQNKIYKFRGTGKAWDW--EVDIENGNYDYLMYADIDMDHPEVINELRN 219

AmyH FDGTDWDQSRSL-SRIYKFKSTGKAWDS--EVSGENGNYDYLMYADVDFEHPEVRQEMKN 245

AmyZ1 FDGTDWDQSRGL-NRIYQFQGTGKAWDY--EVDTENGNYDYLMFADVDYDHPDVVNEMKN 237

BCA FDGTDWDEGRKL-NRIYKFRGIGKAWDW--EVSSENGNYDYLMYADLDFDHPDVANEMKK 244

AmyC FDGTDWDQSRSL-SRIFKFRGTGKAWDW--EVSSENGNYDYLMYADIDYDHPDVVNEMKK 250

BAA FDGADWDESRKI-SRIFKFRGEGKAWDW--EVSSENGNYDYLMYADVDYDHPDVVAETKK 214

BYXA FDGADWDESRKI-SRIFKFRGEGKAWDW--EVSSENGNYDYLMYADVDYDHPDVVAETKK 245

BLA FDGTDWDESRKL-NRIYKFQ--GKAWDW--EVSNEFGNYDYLMYADIDYDHPDVAAEIKR 214

AmyN FDGTDWDESRKL-NRIYKFQ--GKAWDW--EVSNENGNYDYLMYADIDYDHPDVTAEIKR 243

AmyBN10 FNGSD----------NQLY--PGKWWGN-----TFH--FPYLMGEDVDYNRFEVQQEMKA 231

Amy13A FDGVDWDEKTKT-SGKFLF--EGKSWDY-----TFIRDDDYLMGADVDYENLEVQEDVTN 220

**CSR V**

AmyJM WGKWMSDTLDADGYRLDAIKHISHEFINEFIRQIKEHRDG-NFYMVGEYWNEDIEACGNF 275

BHA WGEWYTNTLNLDGFRIDAVKHIKYSFTRDWLTHVRNATGK-EMFAVAEFWKNDLGALENY 278

BliAmy WGTWYANELQLDGFRLDAVKHIKFSFLRDWVNHVREKTGK-EMFTVAEYWQNDLGALENY 273

AmyB WGQWIINNIDFDGFRLDAVKHIDYRFIDKWMSAVQNSSNR-DVFFVGEAWVEDVDDLKGF 392

AmyA WARWVMEQTHCDGFRLDAVKHIPAWFYKEWIEHVQAVAPK-PLFIVAEYWSHEVDKLQTY 277

AmyII WAEWFVETTGVCGFRLDAIKHIDSFFMSNFIRDMKEKYGK-DFYVFGEFWNGDEKTNNDY 275

Amy1 WGKWCLDTTKVDGFRIDAIKHISTWFFPEWIDALERHAGK-DLFMVGEYWYNDINTLLWY 274

Amy63 WGEWYINMTGVDGFRMDAVKHIKYQYLQEWIDHLRWKTGK-ELFTVGEYWNYDVNQLHNF 297

AmyK38 WGSWFTDELDLDGYRLDAIKHIPFWYTSDWVRHQRNEADQ-DLFVVGEYWKDDVGALEFY 273

AliC WGKWFVNTLNLDGVRLDAVKHIKFDYMSSWLSSVKSTTGKSNLFAVGEYWNTSLGALENY 277

Amy WGTWYVNTTNIDGFRLDAVKHIKYSFFPDWLTYVRNQTGK-NLFAVGEFWSYDVNKLHNY 307

AVA WGKWYVNTTNVDGFRLDAVKHIKYSFFPDWLSYVRNQTGK-NLFAVGEFWSYDVNKLHNY 307

AFA WGKWYVTTTNIDGFRLDAVKHIKYSFFPDWLSYVRTQTQK-PLFAVGEFWSYDISKLHNY 277

AmyS WGKWYVNTTNIDGFRLDAVKHIKFSFFPDWLSYVRSQTGK-PLFTVGEYWSYDINKLHNY 276

AmyMK-716 WGKWYVNTTNIDGFRLDAVKHIKFSFFPDWLSYVRSQTGK-PLFTVGEYWSYDINKLHNY 310

AmyTSAS1 WGKWYVNTTNIDGFRLDAVKHIKFSSFPDWLSYVRSQTGK-PLFTVGEYWSYDINKLHNY 273

Gt-Amy WGKWYVNTTNIDGFRLDAVKHIKFSFFPDWLSYVRSQTGK-PLFTVGEYWSYDINKLHNY 310

LAMY WGVWYTNTLNLDGFRIDAVKHIKYSYTRDWLTHVRNTTGK-PMFAVAEFWKNDLAAIENY 278

AmyH WGKWYADSLGLDGFRLDAVKHINHSYLKEWVTSVRQTTGK-EMFTVAEYWKNDLGAINDY 304

AmyZ1 WGSWYTNELNLDGFRLDAVKHIKHSFLKDWVSHVRSTTGK-EMFTVAEYWKNDVGELQDY 296

BCA WGTWYAKELNLDGFRLDAVKHIDHEYLRDWVNHVRQQTGK-DMFTVAEYWQNDIQTLNNY 303

AmyC WGVWYANEVGLDGYRLDAVKHIKFSFLKDWVDNARAATGK-EMFTVGEYWQNDLGALNNY 309

BAA WGIWYANELSLDGFRIDAAKHIKFSFLRDWVQAVRQATGK-EMFTVAEYWQNNAGKLENY 273

BYXA WGIWYANELSLDGFRIDAAKHIKFSFLRDWVQAVRQATGK-EMFTVAEYWQNNAGKLENY 304

BLA WGTWYANELQLDGFRLDAVKHIKFSFLRDWVNHVREKTGK-EMFTVAEYWSYDLGALENY 273

AmyN WGTWYANELQLDGFRLDAVKHIKFSFLRDWVNHVREKTGK-EMFTVAEYWQNDLGALENY 302

AmyBN10 WGEWIINHVGFSGFRMDAIAHVDTDFTRDWINHVQWATSE-DVFFVAEAWVSDI---NGY 287

Amy13A WGNWIINEIGFDGFRLDAVKHIDPRFINQFINDVQKSSSK-DLFFVGEAWIEDINTLAKF 279

**CSR III**

**CSR II**

AmyJM LEATDY-QLDLFDVQLHYKLHRISEEGRDADLSTLFEGTLVEEFPMNAVTFVDNHDSQPG 334

BHA LNKTNW-NHSVFDVPLHYNLYNASNSGGNYDMAKLLNGTVVQKHPMHAVTFVDNHDSQPG 337

BliAmy LNKTNF-NHSVFDVPLHYQFHAASTQGGGYDMRKLLNGTVVSKHPVKAVTFVDNHDTQPG 332

AmyB LDTVGNPDLRVFDFPLRSFFVDMLNGAYMADLRNA-GLVNSPGYENRAVTFVDNHDTDRD 451

AmyA IDQVDG-KTMLFDAPLQMKFHEASRQGAEYDMRHIFTGTLVEADPFHAVTLVANHDTQPL 336

AmyII LATTGH-RFDLIDVRLHQNLFEASKEKAHYDLRQIFNHTLVKNQPNSAVTFVDNHDTQRG 334

Amy1 VDAVRG-KMSVFDVPLHYNFHQASKSGGNYDMRRILDGTMMQQRPTHAVTFVENHDSQPL 333

Amy63 ITKTSG-SMSLFDAPLHMNFYNASKSGGSYDMRQIMDGTLMKDNSVKAVTLVENHDTQPL 356

AmyK38 LDEMNW-EMSLFDVPLNYNFYRASQQGGSYDMRNILRGSLVEAHPMHAVTFVDNHDTQPG 332

AliC ENKTNW-SMSLFDVPLHMNFQAAANGGGYYDMRNLLNNTMMKNHPIQAVTFVDNHDTEPG 336

Amy ITKTNG-SMSLFDAPLHNNFYTASKSSGYFDMRYLLNNTLMKDQPSLAVTLVDNHDTQPG 366

AVA ITKTNG-AMSLFDAPLHNNFYIASKSSGYFDMRYLLNNTLMKDQPALAVTLVDNHDTQPG 366

AFA ITKTNG-SMSLFDAPLHNNFYIASKSGGYFDMRTLLNNTLMKDQPTLAVTLVDNHDTEPG 336

AmyS IMKTNG-TMSLFDAPLHNKFYTASKSGGTFDMRTLMTNTLMKDQPTLAVTFVDNHDTEPG 335

AmyMK-716 ITKTNG-TMSLFDAPLHNKFYTASKSGGAFDMRTLMTNTLMKDQPTLAVTFVDNHDTEPG 369

AmyTSAS1 ITKTNG-TMSLFDAPLHSKFYTASKSGGAFDMRTLMTNTLMKDQPTLAVTFVDNHDTEPV 332

Gt-Amy ITKTNG-TMSLFDAPLHSKFYTASKSGGAFDMRTLMTNTLMKDQPTLAVTFVDNHDTEPV 369

LAMY LNKTSW-NHSVFDVPLHYNLYNASNSGGYFDMRNILNGSVVQKHPIHAVTFVDNHDSQPG 337

AmyH LAKTGY-THSVFDVPLHYNFQAAGNGGGYYDMRNILKGTVVEQHPTLAVTIVDNHDSQPG 363

AmyZ1 LSDVGY-NHSLFDAPLHYNFYDASRSSGNYDMRNLLNGTLVASQPTKAVTLVENHDSQPG 355

BCA LVKVNY-NQSVFDAPLHYNFHYASTGNGNYDMRNILEGTVVANHPTLAVTLVENHDSQPG 362

AmyC LAKVNY-NQSLFDAPLHYNFYAASTGGGYYDMRNILNNTLVASNPTKAVTLVENHDTQPG 368

BAA LNKTSF-NQSVFDVPLHFNLQAASSQGGGYDMRRLLDGTVVSRHPEKAVTFVENHDTQPG 332

BYXA LNKTSF-NQSVFDVPLHFNLQAASSQGGGYDMRRLLDGTVVSEHPEKAVTSVENHDTQPG 363

BLA LNKTNF-NHSVFDVPLHYQFHAASTQGGGYDMRKLLNGTVVSKHPLKSVTFVDNHDTQPG 332

AmyN LNKTNF-NHSVFDVPLHYQFHAASTQGGGYDMRKLLNGTVVSKHPVKAVTFVDNHDTQPG 361

AmyBN10 LDAVNTPHLRAFDFNLREDFVALSSGSK--DMRWW-GGLVNSQHRDRAVTFVDNHDTSRA 344

Amy13A LDTVNNDDLKVFDFPLRSSFEDMINGTDMRTLQYT-GLVNKEGYKNRAVTFVNNHDTNRD 338

**CSR IV**

AmyJM ESLES-WVQDWFKQSAYALVLLRADGYPCVFYGDYFGIDGEDPIE----------D-KRE 382

BHA ESLES-FVQEWFKPLAYALILTREQGYPSVFYGDYYGIP---TH--------SVPA-MKA 384

BliAmy QSLES-TVQTWFKPLAYAFILTREAGYPQIFYGDMYGTKGASQR--------EIPA-LKH 382

AmyB EGSYT-VSIYSRKYQAYAYILTRAEGVPTVYWKDYYIWE------------------MKE 492

AmyA QALEA-PVEPWFKPLAYALILLRENGVPSVFYPDLYGASYEDSGENGETCRVDMPV--IN 393

AmyII QALES-TIDEWFKPAAYALILLRQTGLPCIFYGDYYGISGEFAQQ-----------DFQD 382

Amy1 QALES-VVEPWFKPLAYAIILLRQEGYPCVFHADYYGAEYEDWGKDGNRYNIFMPS-HRW 391

Amy63 QALES-TVDWWFKPLAYAFILLREEGYPSVFYADYYGAQYSDKGH-------DINMVKVP 408

AmyK38 ESLES-WVADWFKPLAYATILTREGGYPNVFYGDYYGIP---ND--------NISA-KKD 379

AliC QALQS-WVSDWFKPLAYATILTRQEGYPCVFYGDYYGIP---SQ--------SVSA-KST 383

Amy QSLQS-WVEPWFKPLAYAFILTRQEGYPCVFYGDYYGIP---KY--------NIPG-LKS 413

AVA QSLQS-WVEPWFKPLAYAFILTRQEGYPCVFYGDYYGIP---KY--------NIPG-LKS 413

AFA QSLQS-WVEPWFKPLAYAFILTRQEGYPCVFYGDYYGIP---KY--------NIPA-LKS 383

AmyS QALQS-WVDPWFKPLAYAFILTRQEGYPCVFYGDYYGIP---QY--------NIPS-LKS 382

AmyMK-716 QALQS-WVDPWFKPLAYAFILTRQEGYPCVFYGDYYGIP---QY--------NIPS-LKS 416

AmyTSAS1 QALQS-WVDPWFKPLAYAFILTRQEGYPCVFYGDYYGIP---QY--------NIPS-LKS 379

Gt-Amy QALQS-WVDPWFKPLAYAFILTRQEGYPCVFYGDYYGIP---QY--------NIPS-LKS 416

LAMY EALES-FVQSWFKPLAYALILTREQGYPSVFYGDYYGIP---TH--------GVPS-MKS 384

AmyH QSLES-TVANWFKPLAYATIMTRGQGYPTLFYGDYYGTKGTTNR--------EIPN-MSG 413

AmyZ1 QALES-VVQSWFKPLAYAFILTREAGYPSVFYGDYYGTKGNSGY--------EIPN-LSQ 405

BCA QSLES-VVSPWFKPLAYAFILTRAEGYPSVFYGDYYGTKGNSNH--------EIPA-LKD 412

AmyC QSLES-TVQPWFKPLAYAFILTRSGGYPSVFYGDMYGTKGTTTR--------EIPA-LKS 418

BAA QSLES-TVQTWFKPLAYAFILTRESGYPQVFYGDMYGTKGTSPK--------EIPS-LKD 382

BYXA QSLES-TVQTWFKPLAYAFILTRESGYPQVFYGDMYGTKGTSPK--------EIPS-LKD 413

BLA QSLES-TVQTWFKPLAYAFILTRESGYPQVFYGDMYGTKGDSQR--------EIPA-LKH 382

AmyN QSLES-TVQTWFKPLAYAFILTREAGYPQIFYGDMYGTKGASQR--------EIPA-MKH 411

AmyBN10 GNPYGMPQVINYKNQAYAYILLREHGVPTVFARDYDEFG------------------MAA 386

Amy13A GK--D-PGIYKRKYQAYAYILTREHGIPVVYWKDYYIYN------------------MKN 377

**CSR VII**

AmyJM AIDPLIYARYHRAYGEQDDYFDH-PATIGWVRRGVEEIEGSGCAVVVTVGDEGE---KRM 438

BHA KIDPILEARQNFAYGTQHDYFDH-HNIIGWTREGNTTHPNSGLATIMSDGPGGE---KWM 440

BliAmy KIEPILKARKQYAYGAQHDYFDH-HNIVGWTREGDSSVANSGLAALITDGPGGT---KRM 438

AmyB GLDKLLTARRYYAYGPGYEVDNNDADIYSYVRSGFPDVAGDGLVLMISDGTSGNVAGKWI 552

AmyA QLDRLILARQRFAHGIQTLFFDH-PNCIAFSRSGTEENP--GCVVVLSNGDDGE---KTL 447

AmyII DIDKLLFLRQAAVYGKEMNYFDN-PNCIGWSYLGDKEHPTS-LAVLINNAHSTA---KRM 437

Amy1 IIDKLLYARKHYAYGPQYNYLDH-WNTIGWTRLGDADHPQ-GMAVIMSDGSEGI---KWM 446

Amy63 YIEELVTLRKDYAYGKQHSYLDH-WDVIGWTREGDAKHPHS-MAVIMSDGPGGS---KWM 463

AmyK38 MIDELLDARQNYAYGTQHDYFDH-WDVVGWTREGSSSRPNSGLATIMSNGPGGS---KWM 435

AliC WLDKQLSARKSYAYGTQHDYLDN-QDVIGWTREGDSAHAGSGLATVMSDGPGGS---KTM 439

Amy KIDPLLIARRDYAYGTQRDYIDH-QDIIGWTREGIDTKPNSGLAALITDGPGGS---KWM 469

AVA KIDPLLIARRDYAYGTQRDYIDH-QDIIGWTREGIDAKPNSGLAALITDGPGGT---KWM 469

AFA KLDPLLIARRDYAYGTQHDYIDS-ADIIGWTREGVAEKANSGLAALITDGPGGS---KWM 439

AmyS KIDPLLIARRDYAYGTQHDYLDH-SDIIGWTREGVTEKPGSGLAALITDGPGGS---KWM 438

AmyMK-716 KIDPLLIARRDYAYGTQHDYLDH-SDIIGWTREGVTEKPGSGLAALITDGPGGS---KWM 472

AmyTSAS1 KIDPLLIARRDYAYGTQHDYLDH-SDIIGWTREGVTEKPGSGLAALITDGPGGS---KWM 435

Gt-Amy KIDPLLIARRDYAYGTQHDYLDH-SDIIGWTREGVTEKPGSGLAALITDGPGGS---KWM 472

LAMY KIDPLLQARQTYAYGTQHDYFDH-HDIIGWTREGDSSHPNSGLATIMSDGPGGN---KWM 440

AmyH SLQPILKARKDFAYGTQHDYINH-QDVIGWTREGVTDRAKSGLATILSDGPGGS---KWM 469

AmyZ1 KLDPLLEARETYAYGIQHDYLDH-QDLVGWTREGDSAHANSGLATLITDRNGGS---KWM 461

BCA KIDPILTARKNFAYGTQRDYFDH-PDVIGWTREGDSVHANSGLATLISDGPGGT---KWM 468

AmyC KIEPLLKARKDYAYGTQRDYIDN-PDVIGWTREGDSTKAKSGLATVITDGPGGS---KRM 474

BAA NIEPILKARKEYAYGPQHDYIDH-PDVIGWTREGDSSAAKSGLAALITDGPGGS---KRM 438

BYXA KLEPILKARKEYAYGPQHDYIDH-PDVIGWTREGDSSAAKSGLAALITDGPGGS---KRM 469

BLA KIEPILKARKQYAYGAQHDYFDH-HDIVGWTREGDSSVANSGLAALITDGPGGA---KRM 438

AmyN KIEPILKARKQYAYGAQHDYFDH-HNIVGWTREGDSSVANSGLAALITDGPGGT---KRM 467

AmyBN10 TLDKLIEARRYFAYGPGHEYSGNTEAVYAYVREGLSNVPGTGLVMLMSGRNWGGQQSFSI 446

Amy13A ELDKLLEVRKYFAYGPGYEVDNNDEDVYSYVREGLEEVEGDGLVLMISDGESGEIISKRI 437

AmyJM FVGEHRAGEEWTDYTNSIDEPVVIDDEGYGTFPVQAG---SVSVWALPAE---------- 485

BHA YVGQNKAGQVWHDITGNKPGTVTINADGWANFSVNGG---SVSIWVKR------------ 485

BliAmy YVGRQNAGETWHDITGNRSDSVVINAEGWGEFHVNGG---SVSIYVQR------------ 483

AmyB NSR--QPDTEFYDLTGHIKEHVTTDSEGYGNFKVIKSEDKGWSIWVPVE----------- 599

AmyA LLGDNYANKTWRDFSGNRDEYVVTNDQGEATFFCNAG---SVSVWVIEDV---------- 494

AmyII FVGKKWAGKTFTDYLGNQNSTIVIDEEGYGSFPVGAE---SVSAYIPQDQ---------- 484

Amy1 EVGKPNT--KFIDLTEHIKEAVYTNEWGWGEFRCLGG---SVSVWVQA------------ 489

Amy63 YTGKPSA--RYVDKLGIRTEEVWTDANGWAEFPVNGG---SVSVWVSVE----------- 507

AmyK38 YVGRQNAGQTWTDLTGNNGASVTINGDGWGEFFTNGG---SVSVYVNQ------------ 480

AliC YVGTAHAGQVFKDITGNRTDTVTINSAGNGTFPCNGG---SVSIWVKQ------------ 484

Amy YVGKKHAGKVFYDLTGNRSDTVTINADGWGEFKVNGG---SVSIWVAKT---SNVTFTV- 522

AVA YVGKKHAGKVFYDLTGNRSDTVTINADGWGEFKVNGG---SVSIWVAKT---SNVTFTV- 522

AFA YVGKQHAGKTFYDLTGNRSDTVTINADGWGEFKVNGG---SVSIWVPKISTTSQITFTV- 495

AmyS YVGKQHAGKVFYDLTGNRSDTVTINSDGWGEFKVNGG---SVSVWVPRKTTVSTIAWSI- 494

AmyMK-716 YVGKQHAGKVFYDLTGNRSDTVTINSDGWGEFKVNGG---SVSVWVPRRPVN-------- 521

AmyTSAS1 YVGKQHAGKVFYDLTGNRSDTVTINSDGWGEFKVNGG---SVSVWVP------------- 479

Gt-Amy YVGKQHAGKVFYDLTGNRSDTVTINSDGWGEFKVNGG---SVSVWVPRKTTVSTIAWPI- 528

LAMY YVGKHKAGQVWRDITGNRSGTVTINADGWGNFTVNGG---AVSVWVKQ------------ 485

AmyH YVGKQNAGEVWKDMTGNNGRLVTINADGWGEFFVNGG---SVSIYTQQ------------ 514

AmyZ1 YVGKRNAGETWVDMTGNRSNQVVINKDGWGEFFVNGG---SVSVYKQK------------ 506

BCA DVGKNNAGEVWHDITGNQTNTVTINKDGWGQFQVSGG---SVSIYVQQ------------ 513

AmyC YVGTSNAGEIWYDLTGNRTDKITIGSDGYATFPVNGG---SVSVWVQQ------------ 519

BAA YAGLKNAGETWYDITGNRSDTVKIGSDGWGEFHVNDG---SVSIYVQK------------ 483

BYXA YAGLKNAGETWYDITGNRSDTVKIGSDGWGEFHVNGG---SVSIYVQK------------ 514

BLA YVGRQNAGETWHDITGNRSEPVVINSAGWGEFHVNGG---SVSIYVQR------------ 483

AmyN YVGRQNAGETWDDITGNRSDSVVINAEGWGEFHVNGG---SVSIYVQR------------ 512

AmyBN10 NSH--QANTTFYDYTGNVAGTVTTNAQGYGSFPVNMTESTGWSVWVPQSSGGPQPGSITL 504

Amy13A NSG--QPNTTFYDYTGHVQGKIITDERGYGDFKVRKSENEGWSVWVPVIKK--------- 486

AmyJM ------------------------------------------------------------ 485

BHA ------------------------------------------------------------ 485

BliAmy ------------------------------------------------------------ 483

AmyB ------------------------------------------------------------ 599

AmyA ------------------------------------------------------------ 494

AmyII ------------------------------------------------------------ 484

Amy1 ------------------------------------------------------------ 489

Amy63 ------------------------------------------------------------ 507

AmyK38 ------------------------------------------------------------ 480

AliC ------------------------------------------------------------ 484

Amy -NNATTTSGQNVYVVANIPELGNWNTANAIKMNPSSYPTWKATIALPQGKAIEFKFIKKD 581

AVA -NNATTVYGQNVYVVGNIPELGNWNIANAIQMTPSSYPTWKTTVSLPQGKAIEFKFIKKD 581

AFA -NNATTVWGQNVYVVGNISQLGNWDPVHAVQMTPSSYPTWTVTIPLLQGQNIQFKFIKKD 554

AmyS -TTR--PWTDEF-VRWTEPRLVAWP----------------------------------- 515

AmyMK-716 ------------------------------------------------------------ 521

AmyTSAS1 ------------------------------------------------------------ 479

Gt-Amy -TTR--PWTGEF-VRWTEPRLVAWP----------------------------------- 549

LAMY ------------------------------------------------------------ 485

AmyH ------------------------------------------------------------ 514

AmyZ1 ------------------------------------------------------------ 506

BCA ------------------------------------------------------------ 513

AmyC ------------------------------------------------------------ 519

BAA ------------------------------------------------------------ 483

BYXA ------------------------------------------------------------ 514

BLA ------------------------------------------------------------ 483

AmyN ------------------------------------------------------------ 512

AmyBN10 RMTKDVGYGYSLFFTGSSPDLTNWGAGIEG--TWTSGHVWEVTIPDPGN--FEWKTRKGP 560

Amy13A ------------------------------------------------------------ 486

**Fig. S2** Multiple sequence alignment of AmyJM and all 27 well-characterized bacterial α-amylases from subfamily GH13_5 using Clustal Omega. The eight conserved regions (CSRs I‒VIII) are indicated by rectangular boxes. The putative catalytic triad (D233, E263, and D330) is shown in orange. Conserved calcium binding site are highlighted in brown. The abbreviations of α-amylase along with their corresponding bacterial origins are listed as follows: AmyJM (*Jeotgalibacillus malaysiensis* D5ᵀ), AmyBN10 (*Alkalimonas amylolytica* N10), AmyTSAS1 (*Bacillus acidicola* TSAS1), BAA (*Bacillus amyloliquefaciens* ATCC 23842), AmyN (*Bacillus licheniformis* NH1), BYXA (*Bacillus* sp. YX), AmyC (*Cytophaga* sp.), Gt-Amy (*Geobacillus thermoleovorans* NP54), Amy1 (*Nostoc* sp. PCC 7119), AmyA (*Salmonella typhimurium* SJW1103), AmyII (*Streptococcus bovis* 148), Amy13A (uncultured bacterium), Amy63 (*Vibrio alginolyticus* 129-63), BLA (*Bacillus licheniformis* ATCC 27811), AmyZ1 (*Pontibacillus* sp. ZY), BliAmy (*Bacillus paralicheniformis* ATCC 9945a), BHA (*Bacillus halmapalus* DSM8723), AliC (*Alicyclobacillus* sp. 18711), LAMY (*Bacillus* sp. KSM-1378), AmyS (*Geobacillus stearothermophilus* DY5), AmyK38 (*Bacillus* sp. KSM-K38), AmyB (*Halothermothrix oren*ii H 168), AFA (*Anoxybacillus flavothermus*), BCA (*Bacillus cereus* GUF8), Amy (*Bacillus* sp. TS-23), AmyH (*Exiguobacterium* sp. DAU5), AmyMK-716 (*Bacillus* sp. MK-716), and AVA (*Anoxybacillus vranjensis* ST4).


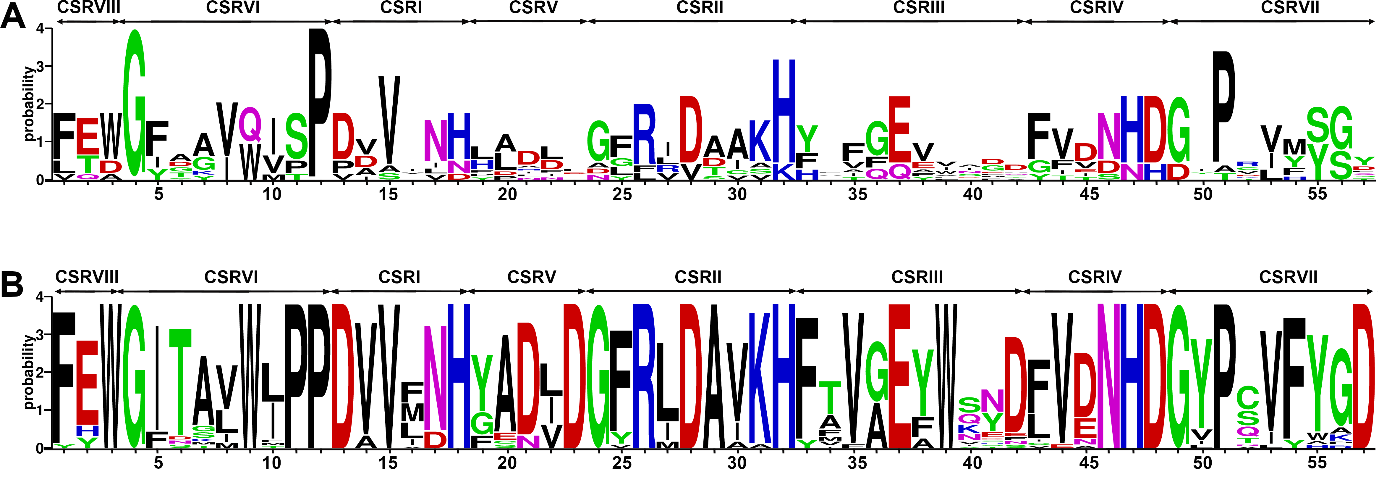


**Fig. S3 A** Comparison of conserved sequence regions (CSRs) between AmyJM and representatives of bacterial α-amylases from the GH13 family using WebLogo3. **B** Comparison of CSRs between AmyJM and all 27 well-characterized bacterial α-amylases belonging to subfamily GH13_5 using WebLogo3.

AmyJM LDAIKHISHEFINEFIRQIKEHRDGNFYMVGEYWNEDIEACGNFLEAT-DYQLDLFDVQL 290

BliAmy LDAVKHIKFSFLRDWVNHVREKTGKEMFTVAEYWQNDLGALENYLNKT-NFNHSVFDVPL 288

AmyB LDAVKHIDYRFIDKWMSAVQNSSNRDVFFVGEAWVEDVDDLKGFLDTVGNPDLRVFDFPL 408

BHA IDAVKHIKYSFTRDWLTHVRNATGKEMFAVAEFWKNDLGALENYLNKT-NWNHSVFDVPL 293

AmyJM HYKLHRISEEGRDADLSTLFEGTLVE--EFPMNAVTFVDNHDSQPGESLESWVQDWFKQS 348

BliAmy HYQFHAASTQGGGYDMRKLLNGTVVS--KHPVKAVTFVDNHDTQPGQSLESTVQTWFKPL 346

AmyB RSFFVDMLNGA---YMADLRNAGLVNSPGYENRAVTFVDNHDTDRDEGSYTVSIYSRKYQ 465

BHA HYNLYNASNSGGNYDMAKLLNGTVVQ--KHPMHAVTFVDNHDSQPGESLESFVQEWFKPL 351

AmyJM AYALVLLRADGYPCVFYGDYFGIDGED--PIEDKREAIDPLIYARYHRAYGEQDDYFD-H 405

BliAmy AYAFILTREAGYPQIFYGDMYGTKGASQREIPALKHKIEPILKARKQYAYGAQHDYFD-H 405

AmyB AYAYILTRAEGVPTVYWKDYYIW---------EMKEGLDKLLTARRYYAYGPGYEVDNND 516

BHA AYALILTREQGYPSVFYGDYYGIP---THSVPAMKAKIDPILEARQNFAYGTQHDYFD-H 407

**Fig. S4** Multiple sequence alignment of *Bli*Amy, AmyB, and BHA surface-binding sites (SBSs) with AmyJM using Clustal Omega. The *Bli*Amy SBS (residues F257 and Y358) are highlighted in gray. The putative AmyJM SBS substituted with aromatic counterparts (residue Y259) is highlighted in red. The abbreviations of α-amylase along with their corresponding bacterial origins are listed as follows: AmyJM (*Jeotgalibacillus malaysiensis* D5ᵀ), BliAmy (*Bacillus paralicheniformis* ATCC 9945a), AmyB (*Halothermothrix oren*ii H 168), and BHA (*Bacillus halmapalus* DSM8723).


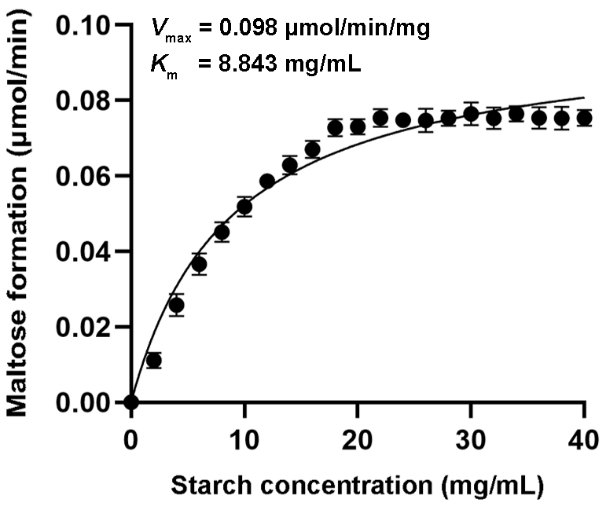


**Fig. S5** Rate vs. substrate concentration plot for AmyJM hydrolysis of soluble starch at different concentrations (2‒40 mg/mL). Values are means ± standard error of triplicate analyses.
